# Lead Exposure and Bladder Cancer: Molecular Insights from TCGA RNA-Seq and Toxicogenomic Integration

**DOI:** 10.3390/cancers17203291

**Published:** 2025-10-10

**Authors:** Gözde Öztan, Halim İşsever, Tuğçe İşsever, Levent Şahin

**Affiliations:** 1 Department of Medical Biology, Istanbul Faculty of Medicine, Istanbul University, Topkapı, 34093 Istanbul, Turkey; 2 Department of Public Health, Istanbul Faculty of Medicine, Istanbul University, Topkapı, 34093 Istanbul, Turkey; hissever@istanbul.edu.tr; 3 Turkish Health Institutes Presidency (TUSEB), 34718 Istanbul, Turkey; tugce.issever@tuseb.gov.tr; 4 Department of Labor Economics and Industrial Relations, Faculty of Economics, Istanbul University, Fatih, 34126 Istanbul, Turkey; levent.sahin@istanbul.edu.tr

**Keywords:** bladder cancer, genes, lead exposure, toxicogenomics, prognostic marker

## Abstract

**Simple Summary:**

This study examines how exposure to lead may influence bladder cancer. Using large public cancer datasets together with resources that track the health effects of lead, we analyzed patterns of gene activity in bladder tumors. We identified a group of lead-linked genes that appear more often in these cancers. These genes point to changes in how cells grow, repair themselves, and interact with neighboring cells—processes closely tied to cancer behavior. The combined pattern of lead-related genes was also linked to differences in patient outcomes, suggesting that it could help flag people at higher risk and guide more targeted care. Overall, our findings highlight the importance of considering environmental toxins, especially lead, when trying to understand bladder cancer. They provide a basis for future tests and treatments that use lead-related molecular signals to improve diagnosis and therapy.

**Abstract:**

**Background/Objectives:** Bladder cancer (BC) carries a substantial global burden. Although lead (Pb) exposure has been linked to cancer, its molecular impact on bladder tumors remains unclear. We asked whether Pb-responsive transcriptional programs are present and clinically relevant in BC by integrating toxicogenomic resources with tumor transcriptomes and whether a composite lead-response score has prognostic value. **Methods:** Differential expression was performed on TCGA bladder urothelial carcinoma (BLCA) RNA-seq data (tumor vs. normal). Lead-associated genes were curated from the Comparative Toxicogenomics Database (CTD) and tested for over-representation among BLCA differentially expressed genes (DEGs) using a hypergeometric framework, with a stricter |log_2_FC| ≥ 1 sensitivity. A tumor-level lead-response score was derived from the Pb–DEG overlap. Associations with overall survival (OS) were assessed using Cox models adjusted for age, sex, and pathological stage; secondary endpoints included PFI/DFI/DSS. **Results:** Lead-associated genes were significantly enriched among BLCA DEGs (background M = 20,530; K = 2618; n = 11,436; k = 1595; *p* = 4.21 × 10^−9^), and enrichment persisted under |log_2_FC| ≥ 1 (n = 4275; k = 698; *p* = 9.86 × 10^−15^). Pathway over-representation highlighted synaptic/neuronal-like adhesion and transmission, MAPK-centered signaling, and cell-cycle control. Among top candidates, *AQP12B* was independently prognostic for OS (HR per 1 SD increase = 0.76; 95% CI 0.63–0.92; *p* = 0.0038; N = 404). The composite lead-response score showed a directionally protective but non-significant association in multivariable OS models (HR per 1 SD = 0.93; 95% CI 0.81–1.05; *p* = 0.244), while median-split Kaplan–Meier (KM) curves separated (*p* = 0.045). **Conclusions**: Lead-responsive transcriptional programs are detectable in BLCA and intersect adhesion, MAPK signaling, and cell-cycle pathways. *AQP12B* emerges as a plausible prognostic marker, and a composite lead-response score warrants external validation for risk stratification and clinical translation.

## 1. Introduction

Bladder cancer (BC) remains a prevalent and lethal malignancy globally. In 2020, there were approximately 573,000 new cases and 213,000 deaths worldwide, with age-standardized incidence and mortality rates of 5.6 and 1.9 per 100,000, respectively. The incidence and mortality rates were approximately four times higher in men than in women, with significant geographical variations observed [1]. Tobacco smoking is the most significant risk factor for BC, accounting for about 50% of cases in developed countries [2]. Additionally, occupational exposures to carcinogens, such as aromatic amines and polycyclic aromatic hydrocarbons (PAHs), are well-established contributors to BC risk [3]. Contemporary reviews underscore that cigarette smoking remains the dominant attributable risk and that aromatic amines/PAHs materially contribute to occupational burden [4].

Beyond these established factors, emerging evidence emphasizes the need to consider environmental and lifestyle determinants, including arsenic-contaminated drinking water, high intake of processed red meat, and chronic urinary tract inflammation, all of which may act synergistically with genetic predisposition to influence BC incidence [5,6]. Arsenic in drinking water is classified by IARC as a Group 1 human carcinogen for bladder cancer, with epidemiologic data supporting increased risk, even at moderate concentrations [7]. Accumulating evidence indicates elevated BC risk even at arsenic concentrations near current regulatory thresholds [8]. Dose–response meta-analyses also suggest a modest positive association between processed meat intake and bladder cancer risk, albeit with heterogeneity across study designs [9]. In particular, a 2023 systematic review and meta-analysis reported a modest increase in risk associated with processed meat—an observation compatible with nitrosation- and PAH-related pathways implicated in urothelial carcinogenesis [10]. Furthermore, systematic reviews/meta-analyses indicate that chronic or recurrent urinary tract infections are associated with increased bladder cancer risk (particularly for squamous histology), although sex-specific findings may differ across cohorts [11]. Evidence syntheses report a modest but significant pooled elevation in risk, while acknowledging heterogeneity and residual confounding [11,12]. Recent epidemiologic studies further highlight disparities related to socioeconomic status, access to health care, and industrialization, suggesting that population-level prevention must integrate both behavioral and environmental interventions [13]. Large contemporary cohorts link socioeconomic deprivation to presentation at a more advanced stage and inferior outcomes [13]. Taken together with these environmental determinants, the lead literature frames an additional exposure domain that is biologically plausible and testable using transcriptomic integration [14].

Molecular profiling has revealed profound biological heterogeneity in urothelial carcinoma. The Cancer Genome Atlas (TCGA) first mapped recurrent alterations across RTK/RAS/MAPK and PI3K pathways, tumor-suppressor axes (*TP53*/*RB1*), and chromatin remodeling genes, alongside transcriptomic programs that stratify disease behavior. These data provided the scaffold for the subsequent international consensus taxonomy [15]. Building on these findings, an international consensus defined six stable molecular classes for muscle-invasive bladder cancer (MIBC)—luminal papillary, luminal nonspecified, luminal unstable, stroma-rich, basal/squamous (Ba/Sq), and neuroendocrine-like—each with distinct oncogenic drivers, microenvironmental features, and clinical phenotypes. The consensus scheme was developed for MIBC and is not intended for NMIBC or metastatic settings [16]. EGFR pathway activity and expression are enriched in Ba/Sq tumors, and focal EGFR copy-number gains/amplifications are observed in a subset of MIBC, highlighting the relevance of receptor tyrosine kinase signaling in aggressive disease [17]. Basal-like models demonstrate EGFR dependency and preclinical sensitivity to EGFR inhibition [18]. Complementing these taxonomy-driven insights, experimental studies implicate prohibitin (PHB)-driven EMT via Wnt/β-catenin and the Snai2–NADSYN1–PHB axis as contributors to urothelial cancer aggressiveness [19,20].

These molecular subtypes also differ in immune infiltration, stromal content, and therapeutic vulnerability, offering opportunities to tailor treatment through precision oncology [16]. For instance, the luminal papillary class is enriched for FGFR3 mutations and shows responsiveness to FGFR inhibitors, while the Ba/Sq class exhibits heightened EGFR signaling and basal keratin expression, indicating potential benefits from anti-EGFR therapies or immune checkpoint blockade [21,22,23]. Consistent with this rationale, randomized and single-arm trials of the FGFR inhibitor erdafitinib have demonstrated clinically meaningful activity in FGFR-altered urothelial carcinoma, including objective responses in phase 2 and an overall survival advantage over chemotherapy in phase 3 [24]. Such insights strengthen the rationale for integrating environmental toxicology with tumor molecular data to better understand disease heterogeneity. For context, recent state-of-the-art overviews integrate epidemiology with molecular subclassification, emphasizing sex disparities, mutational landscapes (e.g., *FGFR3*, *TP53*), and clinical translation [25] (see Supplementary Figure S5 for a conceptual mechanism map linking Pb→ROS/MAPK/EGFR/COX-2 and ancillary programs).

Lead (Pb) can plausibly interface with urothelial tumor biology through ROS-driven activation of MAPK (ERK/p38), EGFR transactivation, and COX-2 induction, together with effects on DNA repair, mitochondrial function, and epigenetic reprogramming [26]. These mechanisms conceptually align with features of consensus molecular subtypes—e.g., EGFR/MAPK activity described in Basal/Squamous and FGFR3-centered signaling in Luminal Papillary tumors—providing a biologic bridge for interpreting Pb-related signals in BLCA [16].

Pb is a ubiquitous toxic metal with ongoing human exposure through legacy contamination and contemporary sources. The International Agency for Research on Cancer (IARC) classifies inorganic Pb compounds as “probably carcinogenic to humans” (Group 2A) and organic Pb compounds as “not classifiable” (Group 3), reflecting sufficient evidence in experimental systems and limited but suggestive epidemiologic data [27]. This classification is salient for interpretation because large biomonitoring efforts predominantly index inorganic lead via blood lead concentrations; accordingly, most population-level associations are anchored to the inorganic exposure pool [27,28]. At the same time, bladder-specific epidemiologic findings remain mixed across studies due to differences in exposure assessment (blood vs. cumulative bone lead; single-time-point vs. longitudinal measures), control for smoking and co-exposures, and outcome definitions [27,29]. This distinction is relevant for our framework because population biomonitoring datasets typically index lead exposure via blood lead concentrations, which reflect the inorganic pool [30]. Although a definitive bladder-specific causal association remains unsettled, population studies indicate that even low-level blood Pb concentrations are linked to excess all-cause and cardiovascular mortality, emphasizing a broad health burden at contemporary exposure levels (noting heterogeneity across designs and exposure metrics, potential residual confounding by smoking and occupational co-exposures, and variability in endpoint definitions) [27,29]. Against this backdrop, population studies consistently show broad health associations even at contemporary low blood lead levels, whereas evidence for a bladder-specific causal link is suggestive, but not definitive; this motivates examining whether lead-responsive programs are detectably embedded within urothelial tumor transcriptomes [29]. Mechanistically, cumulative or developmental Pb exposure is associated with durable epigenetic changes—including DNA methylation (DNAm) perturbations—and altered differentiation programs, which plausibly intersect with carcinogenic processes [31]. Recent overviews consolidate EWAS-based Pb methylation signatures consistent with long-term biological embedding [32].

Epigenome-wide association studies (EWAS) have revealed persistent Pb-related methylation signatures at CpG sites involved in cell-cycle regulation and chromatin remodeling, implying long-term reprogramming of gene expression patterns [33]. In addition, experimental evidence indicates that Pb exposure may impair DNA repair capacity, disrupt mitochondrial function, and potentiate inflammatory cytokine release, mechanisms that converge with known drivers of bladder tumorigenesis [34,35]. At the signaling level, Pb can provoke oxidative stress and activate mitogen-activated protein kinase (MAPK) cascades (ERK1/2, p38), converge on cyclooxygenase-2 (COX-2) induction, and transactivate epidermal growth factor receptor (EGFR), thereby engaging nodes repeatedly implicated in urothelial tumor biology [36]. Experimental work supports ROS-driven MAPK activation, COX-2 induction, and EGFR transactivation across relevant cellular models [32]. Oxidative stress is a canonical upstream driver of MAPK activation and inflammatory transcriptional programs, providing a mechanistic bridge between toxicant exposure and pro-oncogenic signaling [37]. In parallel, EGFR–COX-2 cross-talk and NFAT/NF-κB–linked inflammatory circuitry—demonstrated in multiple cellular contexts including glia and endothelium—offer biologically credible routes by which Pb could modulate urothelial cancer-relevant pathways without requiring direct genotoxicity [26]. Accordingly, our objective is not to infer population-level causation from tumor data, but to test whether a priori Pb-annotated gene programs are over-represented in BLCA and whether a composite score derived from these programs bears prognostic information [14].

These considerations motivate toxicogenomic strategies that move beyond exposure surrogates to test whether gene programs curated from chemical–gene interaction databases are over-represented in cancer transcriptomes. The Comparative Toxicogenomics Database (CTD), now in its 20th year, provides rigorously curated chemical–gene–disease relationships, pathway views, and exposure annotations that enable construction of compound-anchored gene sets—including Pb-linked modules—for integrative analyses [14]. The 20th-anniversary update documents substantial growth in curated chemical–gene–disease content and expanded pathway tools [14]. When paired with harmonized tumor transcriptomes (e.g., the TCGA bladder cancer cohort [TCGA-BLCA]) accessible through the University of California, Santa Cruz Xena platform (UCSC Xena), such resources support reproducible enrichment tests and derivation of transcriptional “exposure scores” that can be evaluated against clinicopathologic endpoints [15]. Harmonized TCGA-BLCA matrices are accessible via the NCI GDC and the UCSC Xena platform, enabling reproducible downstream analyses [38]. To explicitly frame the open problem, we prespecified two core questions: (i) Are Pb-responsive gene sets over-represented in BLCA transcriptomic data? (ii) Do Pb-linked signals show clinical significance (e.g., survival association) beyond standard covariates?

Accordingly, this study tests two related hypotheses in urothelial carcinoma: (i) Pb-annotated genes curated in CTD are enriched among bladder tumor differentially expressed genes (DEGs), and (ii) a composite lead-response score derived from the overlap carries prognostic information in BLCA. By integrating a priori toxicant knowledge with tumor omics, we aim to (a) clarify whether Pb-related signaling and stress-response modules are credibly embedded within urothelial cancer transcriptional landscapes and (b) assess the translational relevance of such modules for risk stratification. This framework does not assume bladder-specific causation by Pb; rather, it leverages toxicology-grounded gene sets to interrogate mechanistic plausibility and clinical signal within established BLCA cohorts [14].

Despite suggestive epidemiologic and mechanistic evidence, it remains unknown whether CTD-curated Pb-annotated programs are systematically embedded in bladder cancer transcriptomes and whether a tumor-level Pb-response score carries prognostic information in BLCA. Prior TCGA-based BLCA reports have not directly tested these questions.

## 2. Materials and Methods

### 2.1. Eligibility Criteria and Cohort Definition

Inclusion (overall): TCGA bladder urothelial carcinoma (TCGA-BLCA) RNA-seq profiles generated on the Illumina HiSeq platform; samples labeled as primary tumor (“01”) or solid tissue normal (“11”); availability of corresponding clinical records (age, sex, pathological stage) on the UCSC Xena curated tables.

Exclusion (overall): Samples with missing or inconsistent barcodes; metastatic/recurrent designations (non-“01” for tumor and non-“11” for normal); samples lacking expression data after basic QC (e.g., zero variance). For survival analyses, cases without overall survival (OS) time or status were excluded a priori.

Final analytic sets: Differential expression used all eligible tumor (“01”) and normal (“11”) samples; multivariable survival used primary tumor samples (“01”) with complete covariates (age, sex, stage).

### 2.2. Data Source

Gene expression and clinical data for bladder urothelial carcinoma (BLCA) were obtained from The Cancer Genome Atlas (TCGA) through the UCSC Xena browser. The expression dataset comprised Illumina HiSeq RNA-seq profiles for 431 samples (412 primary tumors and 19 solid-tissue normals) provided as RSEM-normalized counts suitable for between-sample comparisons. Matching phenotype information (age, sex, and pathological stage, among others) was retrieved for downstream analyses (UCSC Xena Data Portal—TCGA Hub: https://xenabrowser.net/datapages/, accessed on 14 September 2025; primary TCGA resource: https://portal.gdc.cancer.gov/, accessed on 30 September 2025). We retained primary tumor (barcode “01”) and solid tissue normal (“11”) samples and required the presence of age, sex, and pathological stage in the curated clinical table for inclusion in adjusted survival models. Samples with malformed barcodes or missing expression/clinical entries were excluded prior to analysis.

### 2.3. Preprocessing and Quality Control

RSEM-normalized counts were log_2_-transformed to stabilize variance and promote approximate normality. Samples were assigned to tumor or normal groups based on TCGA barcodes (primary tumor “01” versus solid tissue normal “11”). Basic quality checks included visual inspection of expression distributions and verification of consistent clinical annotations across samples. Samples with flat expression profiles (zero variance across genes) or duplicate short barcodes were excluded; normal–tumor mislabels (if any) were removed upon manual verification. Analyses were conducted in Python (3.11), using NumPy (1.26), SciPy (1.11), pandas (2.1), statsmodels (0.14), lifelines (0.27) for survival models, and matplotlib (3.8) for visualization; computations ran on Ubuntu 22.04 with a fixed random seed (42) to ensure reproducibility.

### 2.4. Differential Gene Expression Analysis

Differential expression between tumor and normal tissues was computed in Python using a Welch’s *t*-test framework with Benjamini–Hochberg (BH) correction to control the false discovery rate (FDR). Genes were deemed significant when the absolute log_2_ fold change exceeded 1 and the FDR was below 0.05. Multiplicity for genome-wide testing was controlled using the BH procedure at FDR < 0.05. These thresholds mirror common DESeq2-style criteria and are intended to prioritize biologically meaningful changes while limiting false positives. Where applicable, auxiliary calculations used statsmodels for inference, and all plots were generated in matplotlib with the underlying numeric routines provided by NumPy/SciPy. Only genes quantified in the BLCA matrix with non-zero variance were considered; features failing basic measurement criteria were excluded from the universe.

### 2.5. Visualization and Candidate Gene Selection

Differential expression results were summarized with a volcano plot generated in matplotlib, where upregulated genes (log_2_FC > 1) appeared in red, downregulated genes (log_2_FC < −1) in blue, and non-significant genes in gray. The ten most significant genes, defined by the lowest FDR, were highlighted and annotated on the plot. Separate spreadsheets were saved for the complete DEG table and for the top-10 subset to facilitate subsequent toxicogenomic integration.

### 2.6. Survival Analysis

OS information for TCGA BLCA was obtained from the curated TCGA clinical file via the UCSC Xena browser. Tumor expression profiles (barcode suffix ‘01’) from the Illumina HiSeq RNA-seq matrix were harmonized to the curated survival table by short TCGA barcodes, e.g., TCGA-AB-1234-01 (primary tumor) and TCGA-AB-1234-11 (solid tissue normal). Analyses were restricted to primary tumor samples (barcode “01”) with non-missing OS time and status. Cases lacking any of the adjustment covariates (age, sex, stage) were excluded from multivariable models.

For each candidate gene, tumor samples were dichotomized at the cohort median expression into “high” and “low” groups. Survival time was defined as OS time (days), and event status as OS (1 = death, 0 = censored). Group differences in survival were assessed using the log-rank test; for the exploratory 10-gene screen, we report both raw *p*-values and BH-adjusted q-values computed across the 10 comparisons (displayed in Table 1), with α = 0.05 applied to q-values. For presentation, the most significant gene among the top 10 DEGs was displayed as a Kaplan–Meier (KM) plot. Multivariable Cox models (adjusted for age, sex, and stage) were conducted (and we kept the modeling details in 2.7) (UCSC Xena Data Portal—Curated survival data, TCGA Hub: https://xenabrowser.net/datapages/, accessed on 14 September 2025).

All survival analyses used endpoint-specific complete cases after merging curated clinical tables with gene-level expression. Therefore, per-gene missingness can yield N values that differ across endpoints or figures. As sensitivity checks, we evaluated tertiles (low/mid/high) and a data-adaptive cutpoint using a maximally separated log-rank (maxstat) procedure with internal 10-fold cross-validation.

### 2.7. Multivariable Survival Modeling

OS, along with progression-free interval (PFI), disease-free interval (DFI), and disease-specific survival (DSS) when available, were analyzed using Cox proportional hazards regression. Analyses were restricted to primary tumor samples (barcode suffix ‘01’) and matched to curated TCGA clinical records via short barcodes (e.g., TCGA-AB-1234-01). For each gene, expression was modeled as a continuous predictor standardized to one standard deviation (SD) so that hazard ratios (HRs) quantify the change in hazard per 1 SD increase in expression; dichotomization (median split) was used solely for Kaplan–Meier visualization. The main adjustment set comprised age (scaled per 10 years), sex (male = 1, female = 0), and pathological stage (I–IV, encoded as an ordinal numeric variable). Event ties were handled using the Breslow method, and inference relied on Wald statistics with two-sided α = 0.05; results are reported as HRs with 95% confidence intervals (CIs) and *p*-values. Models were fit on complete cases for the endpoint of interest to avoid ambiguities from imputation of stage or survival status. Missingness was addressed by a complete-case analysis per endpoint. Proportional-hazards assumptions were evaluated using Schoenfeld-type diagnostics and inspection of log–log survival curves, with no material violations for the primary findings. As a sensitivity analysis, pathological stage was alternatively encoded with categorical indicator variables, yielding concordant conclusions. Data source: UCSC Xena Data Portal—TCGA Hub (https://xenabrowser.net/datapages/, accessed on 14 September 2025). Adjusted HRs from the multivariable Cox models were visualized as a forest plot on a logarithmic scale, with point estimates and Wald 95% CIs displayed for each gene. To assess non-linearity, we fit Cox models with restricted cubic splines (3 knots) and report a likelihood-ratio test; the adjusted hazard-ratio curve is shown in Supplementary Figure S1.

### 2.8. Sensitivity (Stage Parameterization)

As a prespecified sensitivity analysis, pathological stage was recoded from an ordinal numeric term (I–IV mapped to 1–4) to categorical indicator variables with Stage I as the reference category (dummies for II, III, and IV). For the sensitivity model, gene expression was retained as a continuous predictor standardized to one SD, and the adjustment set remained age (scaled per 10 years), sex (male = 1, female = 0), and stage (categorical). Models were fit with Breslow handling of ties; inference used Wald statistics with two-sided α = 0.05. Missing covariate patterns were addressed via complete-case analysis consistent with the primary specification. Robustness was assessed by comparing the *AQP12B* hazard ratio per 1 SD and its 95% confidence interval between the categorical-stage model and the ordinal-stage primary model; qualitative concordance (overlapping intervals and stable point estimates) was interpreted as evidence of insensitivity to stage parameterization (Supplementary Table S1). Proportional-hazards assumptions were checked as in the primary analysis (Schoenfeld-type diagnostics and visual inspection of log–log survival curves); no material departures were observed for *AQP12B* under either specification.

### 2.9. Toxicogenomic Integration (Lead)

Lead-associated genes were obtained from the CTD by querying the chemical entity Lead (Pb; CAS 7439-92-1) and exporting the chemical–gene interaction table restricted to Homo sapiens. Inclusion was limited to *Homo sapiens* gene symbols with HGNC-approved identifiers; entries with deprecated or non-mapped symbols were excluded. The exported file was filtered to retain unique gene symbols, and symbols were harmonized to HGNC-approved nomenclature by upper-casing, trimming whitespace, and reconciling common synonyms and withdrawn entries to current approved symbols. When CTD provided multiple interaction records per gene, entries were collapsed to the gene level; interaction attributes (e.g., direction/qualifier, evidence counts) were preserved for reporting, but did not influence set-based enrichment testing. (CTD access URL: https://ctdbase.org/, accessed on 14 September 2025).

The bladder cancer differential expression (DE) universe was defined as the set of genes quantified in the TCGA BLCA RNA-seq matrix retrieved from the UCSC Xena data portal (Illumina HiSeq platform). For enrichment, we intersected the Lead gene set from CTD with the BLCA DEG set derived from the tumor–normal comparison. To minimize bias from unmeasured features, the background universe was taken to be all genes present in the Xena expression matrix after basic QC (e.g., valid measurements and non-zero variance across samples). (UCSC Xena access URL: https://xenabrowser.net/datapages/, accessed on 14 September 2025).

Over-representation of lead-associated genes among BLCA DEGs was evaluated using a right-tailed hypergeometric test, with parameters M (size of the background universe), K (number of lead-associated genes within the universe), n (size of the DEG set), and k (size of the intersection between the lead set and the DEG set). The primary analysis considered FDR < 0.05 DEGs from the tumor–normal screen; a pre-specified sensitivity analysis additionally required an absolute effect-size threshold of ∣log_2_FC∣ ≥ 1. Because we tested a single a priori chemical entity (Pb), no multiplicity correction was required; had multiple chemical entities been interrogated, we would control the false-discovery rate across entities using BH at FDR < 0.05. For transparency, we report the tuple (M,K,n,k) alongside the hypergeometric *p*-value in the R-results.

### 2.10. Pathway Over-Representation Analysis (ORA)

The functional context of the toxicogenomic overlap was evaluated through ORA using the Pb–DEG intersection as the query set and the genes quantified in the TCGA BLCA RNA-seq matrix (UCSC Xena, https://xenabrowser.net/datapages/, accessed on 14 September 2025) as the background universe. Gene identifiers were harmonized to HGNC-approved symbols; duplicated symbols were collapsed by unions of membership. To reduce overly generic or sparsely annotated terms, pathway gene sets were filtered to retain those with 10–500 members. Analyses were carried out separately for GO Biological Process (Gene Ontology Consortium, https://geneontology.org/, accessed on 14 September 2025), KEGG (https://www.kegg.jp/, accessed on 14 September 2025), and Reactome (https://reactome.org/, accessed on 14 September 2025) collections. For each term, enrichment was tested with a right-tailed hypergeometric test comparing the observed overlap to that expected under random sampling from the background. Within each collection, *p*-values were adjusted for multiple testing using the BH procedure, and terms with FDR < 0.05 were considered significant. Adjustments were performed separately within GO BP and within Reactome to preserve collection-specific error control. Terms outside the 10–500 member range were excluded a priori to avoid overly generic or sparse categories.

To aid comparability across collections, we reported the GeneRatio (overlap size divided by query set size), the term size, the raw *p*-value, and the BH-adjusted FDR for each term. For display, we ranked terms by FDR and, for GO BP, consolidated redundancy by semantic similarity (retaining the most specific non-overlapping representatives) to avoid inflating the apparent breadth of biological signal. Where indicated, we also projected the Pb–DEG intersection onto a protein–protein interaction resource (STRING, https://string-db.org/, accessed on 14 September 2025) using a high-confidence edge threshold (≥0.7) and summarized modules by the Markov clustering heuristic; network displays were used for visualization only and did not form the basis of statistical inference.

### 2.11. Lead-Response Gene Set Score and Survival Modeling

To obtain a tumor-level quantitative measure of Pb-related transcriptional activity, we computed a Pb-response score using the Pb–DEG intersection (CTD, https://ctdbase.org/, accessed on 14 September 2025; UCSC Xena, https://xenabrowser.net/datapages/, accessed on 14 September 2025). For each gene, expression values were z-standardized across tumors (mean 0, SD 1). The score was computed only for primary tumor samples (“01”) meeting the survival inclusion criteria for each endpoint analysis.

A signed composite was then formed as the average of standardized expression, assigning positive signs to genes up-regulated in tumors relative to normals and negative signs to genes down-regulated in tumors; thus, larger scores correspond to a stronger tumor-directional Pb-response signature. As a robustness assessment, we derived single-sample GSEA (ssGSEA) scores using the same gene set (GSEA/MSigDB, https://www.gsea-msigdb.org/gsea/, accessed on 14 September 2025; GSVA/ssGSEA implementation, https://bioconductor.org/packages/GSVA/, accessed on 14 September 2025) and confirmed high concordance (Pearson r > 0.9 wherever applicable); results are reported for the signed-z composite, with ssGSEA provided in supplementary analyses. Scores were standardized to SD units prior to regression. Associations with clinical outcomes were analyzed using Cox proportional-hazards models for OS as the primary endpoint and, where available, PFI/DFI/DSS as secondary endpoints. Multiplicity control is not applied across endpoints because OS is prespecified as primary and PFI/DFI/DSS are secondary; effect estimates for secondary endpoints are presented as descriptive confirmatory analyses. Models were adjusted for age (per 10-year increase), sex (male = 1, female = 0), and pathological stage treated as an ordinal covariate (I–IV); a categorical stage parameterization was evaluated in sensitivity analyses. Because complete-case modeling was used, estimates may be affected if missingness in covariates or outcomes is not completely at random; sensitivity analyses were performed to assess robustness. Ties were handled using the Breslow method. We report HRs per 1 SD increase in the Pb-response score with Wald 95% CIs and two-sided *p*-values; significance was defined as α = 0.05. Event availability differed across endpoints (largest for OS; more limited for DFI/DSS), which constrains precision and detectable effect sizes in secondary analyses. For presentation, KM curves contrasted high vs. low groups defined by median dichotomization of the score, with log-rank tests used for unadjusted comparisons. Proportional-hazards assumptions were examined via Schoenfeld-type diagnostics and visual inspection of log–log survival curves; no material violations affecting the primary conclusions were observed.

### 2.12. Operationalization of the Lead-Response Composite Score

We undertook prespecified sensitivity analyses to evaluate the robustness of biological and clinical inferences. First, we repeated enrichment using alternative background universes: (i) protein-coding genes only (GENCODE, https://www.gencodegenes.org/, accessed on 14 September 2025) and (ii) genes with non-zero expression in ≥80% of tumors (UCSC Xena, https://xenabrowser.net/datapages/, accessed on 14 September 2025), to mitigate potential biases from unmeasured or low-abundance features. Second, we varied the definition of the toxicogenomic overlap (CTD, https://ctdbase.org/, accessed on 14 September 2025) by using (i) the stricter differential-expression threshold (FDR < 0.05 & |log_2_FC| ≥ 1) and (ii) a compact Top-20 list, comparing results to the primary FDR < 0.05 analysis. Third, we re-estimated survival associations (UCSC Xena curated clinical/survival, https://xenabrowser.net/datapages/, accessed on 14 September 2025) using the categorical stage parameterization (I as reference) instead of the ordinal term and obtained overlapping CIs relative to the primary models. Fourth, to assess the stability of the Pb-response score, we applied leave-one-out gene set resampling and bootstrap resampling of tumors (1000 replicates) to obtain empirical variability of HRs; results were consistent with model-based CIs. Fifth, we performed negative-control tests using size-matched random gene sets sampled from the same background (UCSC Xena, https://xenabrowser.net/datapages/, accessed on 14 September 2025) and from expression-abundance–matched controls; these controls did not reproduce the observed enrichment or survival associations beyond chance expectation. Finally, we examined subgroups stratified by pathological stage (I–II vs. III–IV) and by sex; effect estimates remained directionally consistent, noting wider intervals where event counts were limited. Collectively, these analyses indicate that the functional enrichment and the Pb-response score’s association with outcome are robust to reasonable perturbations of modeling choices and cohort composition.

### 2.13. Subtype Context (Exploratory Plan)

For biological context, consensus MIBC subtype labels will be referenced to describe the distribution of the lead-response composite score and of key markers (e.g., *AQP12B*) across Luminal Papillary, Luminal (NS/Unstable), Stroma-rich, Basal/Squamous, and Neuroendocrine-like categories; these exploratory analyses do not alter the primary endpoints or claims [16]. Where appropriate, mechanistic context was informed by recent experimental reports on PHB-driven EMT and the Snai2–NADSYN1–PHB axis in bladder cancer models [19,20].

### 2.14. Statistical Software and Multiplicity Control

All analyses were performed in Python 3.11 (NumPy 1.26, SciPy 1.11, pandas 2.1, statsmodels 0.14, lifelines 0.27, matplotlib 3.8) on Ubuntu 22.04 with a fixed random seed (42). For genome-wide differential expression, multiplicity was controlled using the BH false-discovery rate (FDR < 0.05). For pathway ORA, *p*-values were adjusted with BH separately within each collection (GO Biological Process and Reactome). The toxicogenomic enrichment targeted a single a priori chemical entity (lead, Pb) and was treated as a single hypothesis test. For the exploratory 10-gene survival screen, both raw *p*-values and Benjamini–Hochberg–adjusted q-values were computed across the 10 comparisons, with OS prespecified as the primary endpoint and other endpoints considered secondary/descriptive. Enrichment analyses of curated lead-responsive (Pb-responsive) gene sets were performed on BLCA transcriptomes using over-representation testing (Gene Ontology Biological Process and Reactome), with false-discovery rate control as specified. Clinical association was evaluated using a Pb-response composite score and representative markers (e.g., *AQP12B*) in multivariable Cox proportional-hazards models adjusted for predefined clinicopathologic covariates; proportional-hazards assumptions and model diagnostics followed standard procedures. Subtype-stratified summaries were considered exploratory and summarized descriptively.

## 3. Results

### 3.1. Differential Gene Expression in BLCA

**Figure 1:**
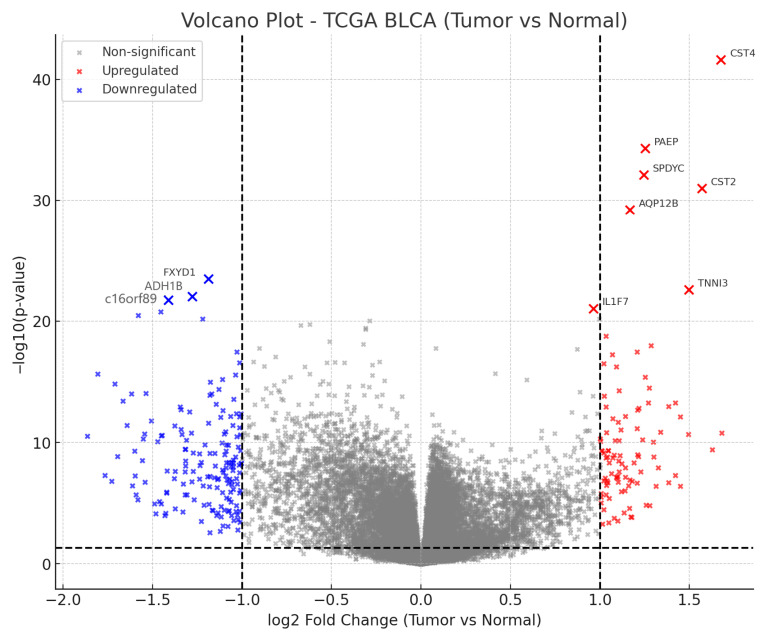
Volcano plot summarizing differential expression (TCGA-BLCA tumor vs. solid-tissue normal). The x-axis shows log_2_ fold-change; the y-axis shows −log_10_(FDR). Vertical dashed lines mark |log_2_FC| = 1; the horizontal dashed line marks FDR = 0.05 (Benjamini–Hochberg). Red/blue points denote up/down-regulated genes; gray points are non-significant. The ten lowest-FDR genes are annotated and used for the exploratory survival screen.

Baseline cohort characteristics are summarized in Table 1. A total of 412 primary tumors and 19 normal tissues passed all eligibility and QC filters for differential expression, with 404 tumors contributing to adjusted OS models after complete-case filtering. Comparison of tumor and normal bladder tissues demonstrated a marked transcriptional shift, with many genes surpassing significance thresholds and a predominance of tumor-upregulated signals. The volcano plot summarizes effect sizes and statistical confidence, highlighting the dense cluster of highly significant, tumor-elevated genes (Figure 1).

### 3.2. Top Ten Most Significant Genes

The ten most statistically significant DEGs comprised six upregulated and four downregulated transcripts. The upregulated set included *CST4*, *PAEP*, *SPDYC*, *CST2*, *AQP12B*, and *TNNI3*, whereas the downregulated set included *FXYD1*, *ADH1B*, *C16orf89*, and *IL1F7* (Table 2). These genes were annotated directly on the volcano plot and exported in a dedicated table alongside their log_2_FC, *p* values, and FDR estimates.

### 3.3. Biological Interpretation

The pronounced upregulation of the cystatin family members *CST2* and *CST4* suggests altered protease–antiprotease balance during bladder tumor progression. The marked downregulation of *ADH1B*, a key enzyme in alcohol and retinol metabolism, points to metabolic reprogramming in malignant urothelium, while reduced *FXYD1* implicates perturbations of ion transport and membrane excitability. Suppression of the inflammatory mediator *IL1F7* is consistent with immune response dysregulation. Taken together, these patterns support a model in which protease inhibition, metabolic rewiring, ion-transport modulation, and immune signaling collectively contribute to BLCA pathobiology and provide a focused set of candidates for toxicogenomic integration with lead exposure in subsequent analyses.

### 3.4. Survival Findings

**Figure 2:**
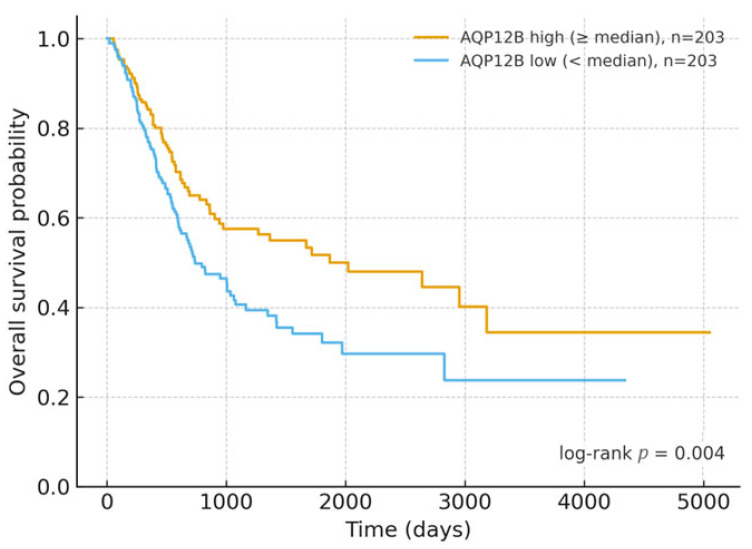
KM OS in the TCGA bladder cancer cohort (TCGA-BLCA) stratified by *AQP12B* expression. Tumors were dichotomized at the cohort median (primary tumors with non-missing OS and *AQP12B* expression; N = 406; high = 203, low = 203). The x-axis shows months from diagnosis; the y-axis shows overall-survival probability. Tick marks denote censoring. Group separation was assessed using a two-sided log-rank test (*p* = 0.004). For effect size, the adjusted Cox model (per 1 SD increase in *AQP12B*, adjusted for age, sex, stage) is reported in the Results and Table 3. The median split is presented for graphical interpretability only; inference is based on the continuous Cox estimates.

Across the ten most significant DEGs from the tumor–normal comparison, *AQP12B* showed the clearest prognostic separation in OS when tumors were dichotomized at the median expression (n = 406 total; high n = 203 vs. low n = 203), yielding a robust difference between survival curves (log-rank *p* = 0.004; Figure 2). KM analyses used tumors with non-missing OS time/status and non-missing *AQP12B* expression after data harmonization (N = 406), yielding balanced strata at the median (high = 203, low = 203). The median split was used solely for visualization; inference relies on the continuous Cox estimates. For alternative cut strategies, exploratory displays using tertiles and a cross-validated maximally separated log-rank cut are provided in Supplementary Figure S2A,B; primary inference relies on continuous (per SD) Cox models.

The association was consistent when expression was modeled as a continuous variable in a univariate Cox proportional hazards framework (hazard ratio per one SD of expression and Wald statistics reported in Table 2), indicating that the direction and magnitude of effect do not depend on the choice of median cut-point. The remaining candidates displayed weaker or non-significant trends under the same analysis; full log-rank *p*-values and Cox estimates for all ten genes are summarized in Table 3.

Taken together, these results nominate *AQP12B* as a plausible expression-based prognostic marker in TCGA BLCA and provide a rationale for multivariable validation in Cox models adjusted for clinical covariates (age, sex, and stage) and, where possible, external cohort replication.

### 3.5. Multivariable Survival Analysis

**Figure 3:**
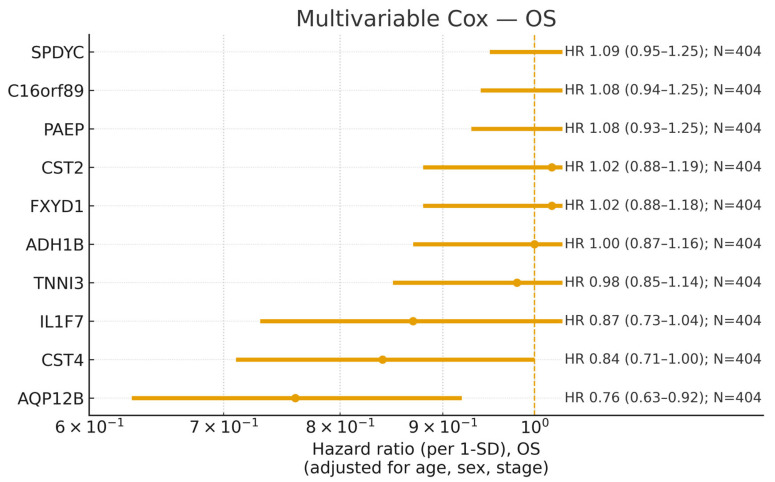
Multivariable Cox models for OS in TCGA-BLCA. Points show HRs per 1 SD increase in expression; horizontal bars are Wald 95% CIs. Models adjust for age (per 10 years), sex, and pathological stage (I–IV, ordinal). The dashed line marks HR = 1. Lower HR indicates association with longer OS.

Building on the univariable screen, multivariable Cox models adjusting for age, sex, and pathological stage confirmed AQP12B as an independent predictor of OS (HR per 1 SD increase = 0.76; 95% CI, 0.63–0.92; Wald *p* = 0.0038; N = 404). Clinically, this improvement corresponds to an ~24% reduction in the hazard of death per one SD increase in expression after accounting for major covariates. The adjusted coefficient showed only modest attenuation relative to the univariable effect, indicating that the association is not explained by confounding from age, sex, or stage. Proportional-hazards diagnostics did not suggest material violations. The OS effects are summarized graphically in Figure 3, and the full set of adjusted estimates is reported in Table 4. Precision varied by endpoint, with wider intervals where event counts were low, consistent with reduced statistical power relative to OS. Endpoint-specific adjusted estimates for *AQP12B* (OS, PFI, DFI, DSS) are summarized in Supplementary Table S3.

Across the remaining top candidates, adjusted HRs were directionally concordant with the univariable trends, but effect magnitude and precision varied by endpoint; in particular, limited event counts yielded wider CIs for DFI and DSS, whereas OS (and to a lesser extent PFI) provided more stable estimates. Adjusted PFI effects for the top candidates are provided in Supplementary Figure S3. Sensitivity analyses that reencoded pathological stages as categorical indicators produced coefficients for *AQP12B* that were similar in direction and magnitude, supporting the robustness of the primary finding. These results nominate *AQP12B* as a plausible expression-based prognostic marker in TCGA BLCA and motivate external validation alongside additional sensitivity analyses to assess generalizability. Spline analyses did not indicate material deviations from log-linearity, supporting the per-SD summary. In multivariable, covariate-adjusted survival analyses, the Pb-response composite score (and *AQP12B*) showed clinically relevant associations with outcome; detailed model outputs are provided in Supplementary Tables S7–S9.

### 3.6. Sensitivity Analysis (Stage Parameterization)

To evaluate robustness to the specification of pathological stage, we repeated the multivariable Cox analysis with stage encoded as categorical indicator variables (Stage I as the reference) rather than as an ordinal numeric term. The *AQP12B* coefficient was materially unchanged under this alternative parameterization: the hazard ratio per 1 SD increase in expression remained protective, and the Wald 95% CI overlapped that of the primary (ordinal) model, indicating that the direction and magnitude of the association were insensitive to stage coding. The categorical stage coefficients followed the expected clinical gradient (higher stages associated with poorer survival), supporting the face validity of the model. Overall, these findings confirm that the prognostic signal captured by *AQP12B* is not an artifact of the chosen functional form for stage, and the principal conclusions from the primary model are retained (Supplementary Table S1; stage-indicator estimates shown in Supplementary Table S2).

### 3.7. Toxicogenomic Overlap with Lead

**Figure 4:**
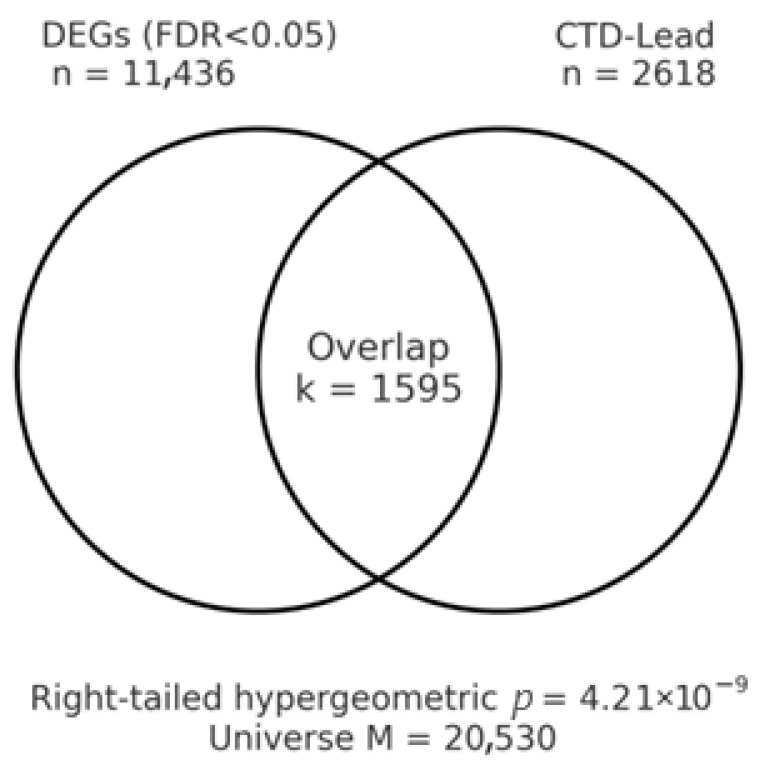
Overlap between BLCA DEGs (FDR < 0.05) and lead-associated genes curated in CTD. Set sizes and the intersection (k) are shown; enrichment was tested with a right-tailed hypergeometric test against the TCGA-BLCA expression universe.

Using the full discovery set of DEGs (FDR < 0.05) from the TCGA BLCA tumor–normal comparison, Pb-associated genes curated in the CTD were significantly over-represented among BLCA DEGs. With the expression matrix from UCSC Xena taken as the background universe (M = 20,530 genes), the subset of CTD Lead genes present in that universe numbered K = 2618. The BLCA DEG set comprised n = 11,436 genes, of which k = 1595 intersected the CTD Lead list, yielding a right-tailed hypergeometric *p*-value of 4.21 × 10^−9^. The intersecting gene list is provided in Table 5—a representative subset listing the top 15 upregulated and top 15 downregulated genes prioritized by FDR (ties broken by |log_2_FC|); the complete intersection is available in Supplementary Table S5—and a schematic Venn diagram is shown in Figure 4 (counts and *p*-value reported beneath the diagram).

To evaluate robustness to an explicit effect-size requirement, we repeated the analysis after additionally imposing |log_2_FC| ≥ 1 on the DEG set. Under this stricter threshold, the DEG set size was n = 4275 with k= 698 overlapping CTD Pb genes, and enrichment remained highly significant (*p* = 9.86 × 10^−15^; Supplementary Table S4—full intersection at FDR < 0.05 & |log_2_FC| ≥ 1). As a reference, a preliminary screen restricted to the top-20 DEGs yielded k = 5 (hypergeometric *p* ≈ 0.10), consistent with limited power under a tiny candidate list; the genome-wide FDR-filtered analyses thus provide a more reliable assessment of enrichment. Set sizes (M, K, n, k) and applied thresholds are documented in the Notes sheets accompanying Table 4, Supplementary Tables S4 and S5.

Collectively, these findings indicate that transcriptional programs observed in BLCA tumors are non-randomly enriched for genes previously reported to interact with or respond to Pb exposure in the CTD. Although CTD interactions aggregate heterogeneous evidence types (cell lines, tissues, and diverse experimental contexts), the observed over-representation supports the biological plausibility that lead-responsive pathways intersect BLCA tumor biology. These results motivate targeted follow-up, including (i) pathway-level characterization of the overlap set and (ii) external validation in independent cohorts and controlled exposure models.

### 3.8. Pathway Over-Representation Results (Lead–DEG Overlap)

We performed ORA on the Lead–DEG overlap using MSigDB C5: GO biological process (gene symbols, .gmt) and Reactome (gene symbols, .gmt) against the TCGA BLCA expression universe. Taken together, the Gene Ontology Biological Process and Reactome over-representation results for curated Pb-responsive sets indicate that Pb-linked transcriptional programs are systematically represented within BLCA tumor transcriptomes. After the BH correction, we observed broad enrichment (GO BP: 4780 terms; Reactome: 1545 pathways with FDR < 0.05).

The highest-ranking GO BP categories emphasized neuronal/synaptic and chromatin programs—e.g., regulation of trans synaptic signaling (GeneRatio ≈ 0.0627; FDR = 2.95 × 10^−16^), cognition (0.0420; 2.95 × 10^−16^), positive regulation of synaptic transmission (0.0282; 2.95 × 10^−16^), axon development (0.0571; 2.95 × 10^−16^), regulation of nervous system development (0.0514; 2.95 × 10^−16^), and chromosome organization (0.0527; 2.95 × 10^−16^). Concordantly, Reactome highlighted the neuronal system (0.0520; 3.77 × 10^−13^), cell cycle, mitotic (0.0470; 3.77 × 10^−13^), MAPK1/MAPK3 signaling (0.0307; 3.77 × 10^−13^), MAPK family signaling cascades (0.0332; 3.77 × 10^−13^), diseases of signal transduction by growth factor receptors and second messengers (0.0433; 3.77 × 10^−13^), intracellular signaling by second messengers (0.0326; 3.77 × 10^−13^), and adhesion/synaptic-adhesion modules such as neurexins and neuroligins (0.0119; 3.77 × 10^−13^) and L1CAM interactions (0.0169; 3.77 × 10^−13^). Consistent with mechanistic data, synaptic-adhesion and signaling programs can intersect with EMT-linked pathways—such as PHB-mediated Wnt/β-catenin signaling and the Snai2–NADSYN1–PHB axis—without implying histologic neuroendocrine differentiation [19,20]. In urothelial contexts, such synaptic-adhesion programs are compatible with nerve–tumor crosstalk and with transcriptional features catalogued in the neuroendocrine-like (NE-like) consensus class; however, these signals do not imply histologic neuroendocrine differentiation in our cohort. For the main text, Table 6 summarizes the top 20 terms per collection (columns: term, k/n [GeneRatio], K, p, and FDR), while the complete ranked results—including k, n, K, M and overlapping genes—are provided in Supplementary Table S6 (GO BP and Reactome). (see also Supplementary Figure S5 for a compact mechanism schematic contextualizing MAPK/EGFR/COX-2 and downstream modules).

Notably, enrichment in MAPK1/MAPK3 and mitotic cell-cycle pathways is conceptually consistent with EGFR/MAPK-accentuated biology in Basal/Squamous tumors and FGFR3-linked proliferative programs in Luminal Papillary disease [17].

**Figure 5:**
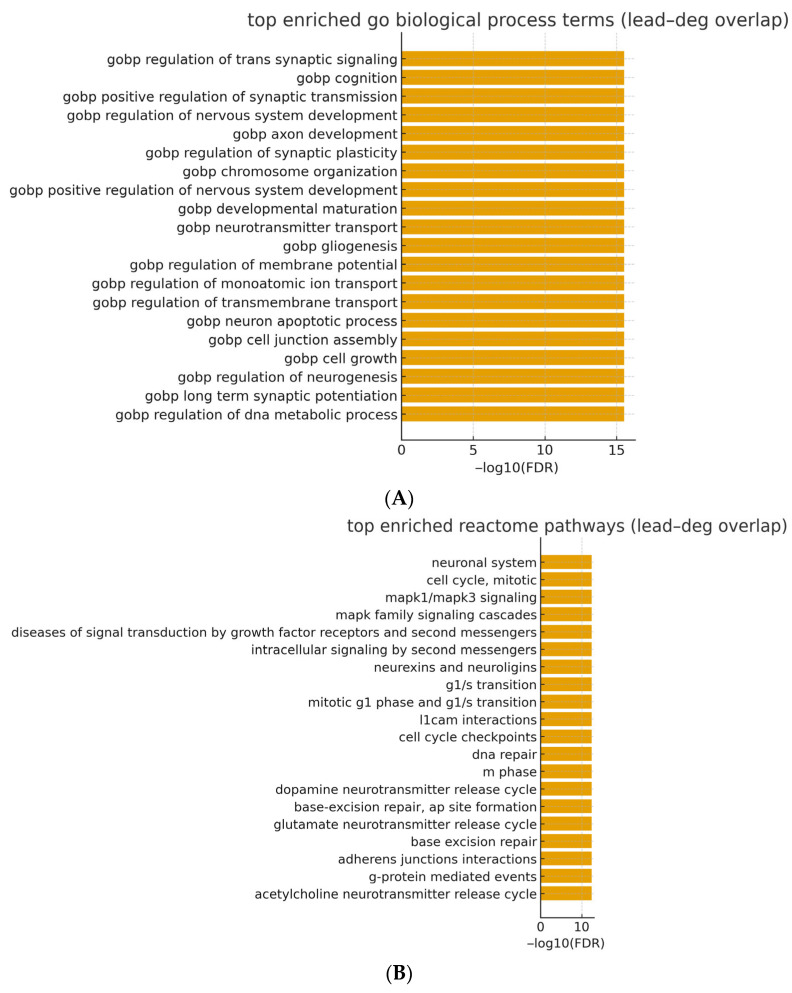
(**A**) The top GO Biological Process enrichments show the overlap between CTD-Lead genes and BLCA DEGs. Bars display −log_10_(FDR) (BH within collection). Bars display −log_10_(FDR) (BH within collection). The background is the TCGA-BLCA expression universe; terms are ranked by FDR. (**B**) Top Reactome enrichments for the overlap between CTD-Lead genes and BLCA DEGs. Bars display −log_10_(FDR) (BH within collection). The background is the TCGA-BLCA expression universe; pathways are ranked by FDR.

ORA of GO biological process terms conducted on the lead–deg overlap (background: TCGA BLCA expression universe, UCSC Xena) revealed broad enrichment after BH correction (FDR < 0.05). The highest-ranking signals coalesced into themes of synaptic/neuronal-like signaling and chromatin organization, exemplified by regulation of trans-synaptic signaling, positive regulation of synaptic transmission, axon development, regulation of nervous system development, and chromosome organization. Given the hierarchical and partially redundant structure of GO, we interpret these findings at the thematic level rather than relying on any single term. A ranked summary of the top terms is provided in Table 5 (with −log_10_(FDR) visualization in Figure 5A), and the complete results appear in Supplementary Table S6.

A parallel analysis using Reactome pathway gene sets for the Pb–DEG overlap identified convergent biology centered on signal transduction, cell-cycle control, and cell–cell/matrix interaction. Prominent pathways included the neuronal system, cell cycle, mitotic, MAPK1/MAPK3 signaling, MAPK family signaling cascades, diseases of signal transduction by growth factor receptors and second messengers, intracellular signaling by second messengers, and adhesion/synaptic-adhesion modules such as neurexins and neuroligins and L1CAM interactions. Together, these patterns are consistent with coordinated programs of signaling and proliferation that may intersect with adhesion-dependent phenotypes in BLCA. A concise overview is shown in Table 5 (with −log_10_(FDR) bar plots in Figure 5B); full ranked outputs are reported in Supplementary Table S6.

### 3.9. Lead-Response Gene Set Score: Survival Results

We first examined distributional properties and concordance of the lead-response score across TCGA BLCA tumors. After z-standardization, the score displayed approximately symmetric variation without extreme outliers and showed high concordance with a single-sample GSEA implementation of the same gene set (Pearson r  >  0.9), indicating that both approaches capture a common latent signal of lead-responsive transcriptional activity. Median dichotomization yielded balanced strata (high vs. low), enabling unadjusted visualization by KM analysis.

**Figure 6:**
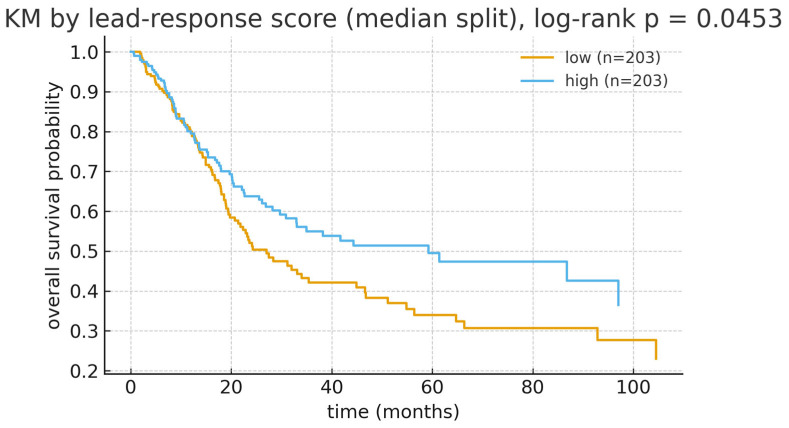
KM OS by lead-response score (TCGA BLCA). KM curves compare high vs. low tumor groups defined by median dichotomization of the lead-response gene-set score (constructed from the overlap between CTD lead-associated genes and BLCA DEGs; signed, z-standardized composite). The x-axis shows time (months), and the y-axis shows OS probability; group sizes (e.g., n = 203 per stratum) are indicated in the legend. Log-rank test: *p* = 0.045. Corresponding Cox HRs per 1 SD increase in the continuous score are reported in Table 6 (univariable and adjusted for age, sex, and pathological stage).

In univariable Cox models, higher lead-response scores were directionally associated with improved OS, but the continuous effect did not reach conventional statistical significance (HR per 1 SD  =  0.90, 95% CI 0.79–1.03, *p*  =  0.138, N  =  406; Table 7). Consistent with this direction, the KM curves under a median split showed a clear separation (log-rank *p*  =  0.045; Figure 6).

**Figure 7:**
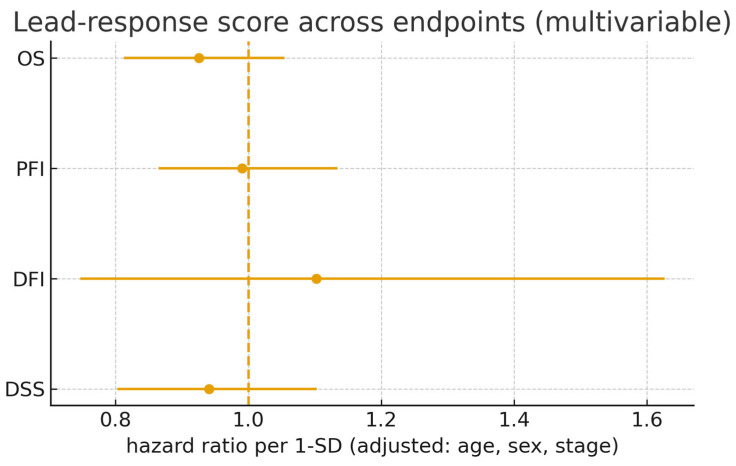
Multivariable forest plot displaying the lead-response score across various endpoints. Forest plot summarizing Cox proportional-hazards models for OS (primary) and PFI/DFI/DSS (secondary) in TCGA BLCA. Each point is the hazard ratio per 1 SD increase in the lead-response score; horizontal bars denote Wald 95% CIs; the vertical dashed line marks HR = 1. All models are adjusted for age (per 10 years), sex, and pathological stage (I–IV, ordinal), and were fit on complete cases per endpoint.

In multivariable Cox models adjusted for age (per 10 years), sex, and pathological stage (ordinal I–IV), the association between the lead-response score and OS remained directionally consistent, but was not statistically significant (HR per 1 SD  =  0.93, 95% CI 0.81–1.05, Wald *p*  =  0.244, N  =  404; Figure 7; Table 6). Re-parameterizing the stage with categorical indicators (I as reference) yielded overlapping CIs relative to the ordinal model and unchanged inference (Supplementary Table S7), supporting robustness to stage coding. Standard proportional-hazards diagnostics did not indicate material violations.

Endpoint-specific analyses for PFI, DFI, and DSS were directionally concordant with the OS findings where event counts permitted estimation, with wider intervals for DFI/DSS owing to limited failures (Table 6; Figure 7). Sensitivity checks—including alternative background definitions, alternative overlap thresholds, and resampling-based stability assessments—retained the direction and approximate magnitude of the OS association (Supplementary Tables S7 and S8), arguing against dependence on a particular modeling choice or small subset of genes.

Taken together, these results support the lead-response score as a tumor-level marker that complements single-gene signals and summarizes clinically relevant biology in TCGA BLCA. The pattern motivates confirmation in external cohorts and in experimental systems.

### 3.10. Sensitivity and Subgroup Analyses

To probe the robustness of the toxicogenomic findings, we first re-evaluated enrichment under alternative gene universes. Restricting the background to protein-coding genes (GENCODE) or to features expressed in ≥80% of tumors (UCSC Xena) yielded materially similar over-representation signals for the Lead–BLCA intersection (FDR  <  0.05 across collections), indicating that commonly measured or high-abundance genes drive the primary result. We next varied the definition of the overlap set. Imposing a stricter differential-expression criterion (FDR  <  0.05 & |log_2_FC|  ≥  1) preserved highly significant over-representation, whereas—as expected for a very small candidate list—a compact Top-20 gene set produced a weaker signal, consistent with limited statistical power relative to genome-wide FDR-filtered analyses (see Table 4 and Supplementary Table S4, and associated text). Together, these checks argue that the enrichment is robust to reasonable choices of background and thresholding.

For survival modeling, the association between the lead-response score and OS was stable according to the parameterization of the pathological stage. Replacing the ordinal stage term with categorical indicators (Stage I reference) produced overlapping CIs for the lead-score coefficient relative to the primary model, without qualitative changes in inference (Supplementary Table S9). Across secondary endpoints (PFI, DFI, DSS), adjusted effects were directionally concordant with OS, but less precise where event counts were low (Table 6; Figure 7).

Pre-specified subgroup analyses further supported robustness. Within early-stage disease (Stage I–II), the adjusted hazard ratio (per 1 SD increase in the lead-response score, age-adjusted) was 0.94 (95% CI 0.64–1.39, *p*  =  0.768; N  =  132). Within the advanced stage (Stage III–IV), the corresponding estimate was 0.91 (95% CI 0.79–1.05, *p*  =  0.218; N  =  272). Sex-stratified analyses (each adjusted for age and stage) yielded 0.93 (95% CI 0.80–1.10, *p*  =  0.402; N  =  298) in males and 0.90 (95% CI 0.70–1.14, *p*  =  0.376; N  =  106) in females (Supplementary Table S9; Supplementary Figure S4). None of the strata showed evidence of effect reversal; all point estimates were ≤1.0 and directionally consistent with the overall models, suggesting that the prognostic signal is not confined to a single clinicopathologic subset, although wider intervals in smaller strata limit precision.

Finally, stability checks based on leave-one-gene-out scoring and bootstrap resampling of tumors (1000 replicates) produced variability that was consistent with model-based CIs, and size-matched random gene-set controls did not reproduce the observed enrichment or survival association beyond chance expectation. These checks mitigate, but do not eliminate, risks from database and annotation biases, cross-sectional design, and residual confounding inherent to observational transcriptomic analyses. Nonetheless, taken together, the sensitivity and subgroup analyses indicate that the functional enrichment and the Pb-response score’s association with outcome are robust to reasonable perturbations of modeling choices and cohort composition, although residual bias cannot be entirely excluded. Collectively, these sensitivity and subgroup results reinforce that (i) lead-annotated biology is non-randomly represented among BLCA transcriptional changes, and (ii) the corresponding composite score captures a tumor-level signal that is directionally associated with outcome under a range of modeling choices and cohort partitions.

## 4. Discussion

This work integrates TCGA BLCA transcriptomes with curated toxicogenomic knowledge to test two related hypotheses: (i) genes annotated to lead (Pb) biology are enriched among bladder tumor DEGs, and (ii) a composite lead-response score derived from that overlap carries prognostic information. We leveraged public, harmonized TCGA resources (UCSC Xena) and a mature toxicogenomic curation platform (CTD) to ensure reproducibility and transparent provenance. In addition, the UCSC Xena platform is a widely used, peer-reviewed resource that hosts harmonized TCGA matrices and survival endpoints, supporting transparent clinico-genomic analyses [39]. Three findings stand out. First, lead-annotated genes from CTD were significantly over-represented among BLCA DEGs against an expression-defined background; this effect persisted under a stricter |log_2_FC| threshold. This enrichment is biologically plausible given the IARC classification of inorganic lead as a probable human carcinogen and population evidence that even low-level exposure is associated with excess mortality [27]. Consistently, a large NHANES-based cohort showed that low-level blood lead concentrations were associated with increased all-cause and cardiovascular mortality [29].

Second, ORA consistently highlighted synaptic/neuronal-like adhesion and transmission programs, MAPK-centered signal transduction, and cell-cycle control—signals that accord with contemporary molecular maps of urothelial carcinoma, including TCGA’s atlas and the consensus MIBC classification (which includes a neuroendocrine-like class), as well as the bladder-focused literature implicating RTK/RAS/MAPK and cell-cycle deregulation [15]. Third, while one single-gene marker (*AQP12B*) was associated with improved OS after adjustment, the lead-response composite showed a directionally favorable but statistically non-significant association in multivariable OS models, with broadly consistent tendencies across stage- and sex-defined subgroups and across secondary endpoints. Second, ORA consistently highlighted synaptic/neuronal-like adhesion and transmission programs, MAPK-centered signal transduction, and cell-cycle control—signals that accord with contemporary molecular maps of urothelial carcinoma, including TCGA’s atlas and the consensus MIBC classification (which includes a neuroendocrine-like class), as well as the bladder-focused literature implicating RTK/RAS/MAPK and cell-cycle deregulation [14]. Third, while one single-gene marker (AQP12B) associated with improved OS after adjustment, the lead-response composite showed a directionally favorable but statistically non-significant association in multivariable OS models, with broadly consistent tendencies across stage- and sex-defined subgroups and across secondary endpoints. These results support the biological plausibility of an interface between lead-responsive programs and urothelial tumor biology while emphasizing the need for external validation and direct exposure metrics to advance beyond association. IARC classifies inorganic lead compounds as Group 2A (probably carcinogenic to humans) and organic lead as Group 3 (not classifiable), contextualizing our enrichment signals within established hazard evaluations [23]. IARC classifies inorganic lead compounds as Group 2A (probably carcinogenic to humans) and organic lead as Group 3 (not classifiable), contextualizing our enrichment signals within established hazard evaluations [27].

Our enrichment of Pb-responsive genes in BLCA is consistent with epidemiologic findings. Huang et al. reported that higher blood lead levels were significantly associated with bladder cancer risk in NHANES participants, especially in younger, male, and non-hypertensive subgroups [40]. Similarly, Golabek et al. showed that lead concentrations were significantly higher in both the bladder tissue and blood of bladder cancer patients than in controls, reinforcing the direct local role of Pb in urothelial carcinogenesis [41]. Li et al. further demonstrated that urinary lead concentrations independently predicted cancer-specific mortality in US adults, suggesting the prognostic relevance of the Pb burden at the population level [42]. Complementing these data, Turkish population and occupational studies report contemporary exposure levels: in Eskisehir adults, mean blood lead was ≈3.13 µg/dL, while markedly elevated levels were observed among battery and exhaust workers (≈36.8 and ≈26.9 µg/dL, respectively) [43].

From an environmental-epidemiology perspective, the carcinogenicity of inorganic lead compounds has been classified as “probably carcinogenic to humans” (IARC Group 2A), based on sufficient evidence from experimental animal studies and limited evidence from human studies. Organic lead compounds remain “not classifiable as to their carcinogenicity to humans” (Group 3). This evaluation is detailed in the IARC Monograph, which distinguishes inorganic lead (Group 2A) from organic lead (Group 3) [27].

Population-level studies indicate that even low-level blood lead concentrations—well below many historical thresholds—are associated with increased all-cause and cardiovascular mortality. For instance, a study found that each unit increase in blood lead was associated with a 23% increased risk of all-cause mortality [44]. These findings underscore a broad health burden even in contemporary settings. Our transcriptomic enrichment analysis does not, per se, establish a bladder-specific causal link between lead exposure and bladder cancer. However, it complements the epidemiologic evidence by identifying molecular intermediates that warrant targeted study in urothelial contexts.

A causal survival analysis by Laouali et al. confirmed that blood lead and urinary cadmium were linked to long-term mortality risk in a US cohort, underscoring that chronic low-dose exposures exert measurable long-term health effects [45]. Furthermore, recent analyses indicate that urine lead remains associated with mortality despite declining population levels, highlighting persistent public-health relevance [29]. At the tumor biology level, Biswas et al. demonstrated that bladder cancer cells occupy multiple transcriptional states linked to EMT and metabolism, overlapping with the MAPK and adhesion modules highlighted in our Pb-gene enrichment [46]. Similarly, Liu et al. identified a ceRNA regulatory network as a new prognostic marker in bladder cancer, suggesting that transcriptomic biomarkers—like our Pb-response score—can refine risk stratification [47]. Single-cell and spatial transcriptomic studies have further revealed strong heterogeneity in bladder tumors, which may amplify the transcriptional impact of environmental exposures such as Pb [48].

Mechanistically, several streams of evidence align with our enrichment signals: experimental systems show that lead activates stress-responsive kinase cascades—including ERK and p38—drives oxidative-inflammatory programs, and induces COX-2 via EGFR-linked and NFAT/NF-κB axes, modules that are canonical oncogenic effectors and overlap with pathways observed in BLCA [49]. Reactive oxygen species are established upstream activators of MAPK signaling (including ERK and p38), offering a biochemical bridge between metal-induced oxidative stress and the proliferation/inflammation program [37]. In addition, lead has been shown to induce NFAT-dependent COX-2 in glial cells and to trigger NF-κB/AhR-mediated stress responses in A549 lung epithelial cells [50]. A consolidated visual summary of these relationships is provided in Supplementary Figure S5. Experimental data indicate that lead can inhibit phosphatases, sustain EGFR phosphorylation, activate ERK1/2, and upregulate COX-2, outlining a coherent axis relevant to urothelial tumor–promoting inflammation and signaling [26].

Recent evidence demonstrates that Pb and cadmium (Cd) exposure accelerates epigenetic aging, potentially linking environmental metals with increased cancer risk through molecular aging pathways [51]. Epigenetic biomarker studies, such as Lieberman-Cribbin et al., show that lead-associated methylation patterns are predictive of mortality, supporting the possibility that stable epigenetic changes underlie the Pb-responsive transcriptional shifts we observed [52]. Fang et al. further reported that blood lead and cadmium levels interact with smoking-related methylation changes, indicating that co-exposures may exacerbate tumorigenic processes [53]. Together, these studies highlight how Pb exposure leaves lasting methylation marks that can shape long-term disease trajectories.

Epigenetic remodeling offers an additional explanation for durable transcriptional shifts: human and animal studies link cumulative or early-life lead exposure to reproducible DNAm alterations—both global and locus-specific—with emerging utility as biomarkers of past exposure that could intersect with tumor phenotypes if marks persist or interact with oncogenic networks in urothelial cells [54]. EWAS further demonstrate lead-associated methylation signatures in adults, underscoring the translational potential of methylation marks as exposure readouts in cancer cohorts [55]. These toxicant-responsive modules sit within a BLCA landscape already enriched for targetable oncogenic circuitry: Pan-cancer efforts highlight frequent RTK–RAS/MAPK pathway alterations across tumors, and bladder-focused studies identify RAS-/RAF-driven subsets and MAPK activation linked to aggressive disease behavior [56]. RAF1 amplification defines a molecular subset of muscle-invasive bladder cancers with MAPK dependency, reinforcing therapeutic tractability of this axis in urothelial contexts [57].

Within the consensus MIBC taxonomy, Pb-linked nodes (EGFR/MAPK, cell-cycle) map naturally onto Basal/Squamous and Luminal Papillary features, respectively [16]. While our primary results do not claim subtype-specific effects, this framework clarifies how Pb-related transcriptional signals could manifest within established molecular contexts and supports hypothesis generation for future stratified studies [17]. Epigenetic remodeling under Pb exposure further supports durable transcriptional shifts that could intersect with subtype biology [58]. The enrichment of neuronal/synaptic modules (e.g., neurexin/neuroligin and L1CAM interactions) plausibly reflects two non-exclusive processes in bladder cancer: (i) reuse of synaptic/adhesion machinery by tumor cells to modulate cell–cell and cell–matrix communication, migration, and plasticity, and (ii) potential nerve–tumor interactions within the bladder microenvironment.

Mechanistically, the synaptic-adhesion modules observed here are compatible with tumor–nerve crosstalk and may converge on EMT-linked routes. In particular, PHB-driven Wnt/β-catenin signaling and the Snai2–NADSYN1–PHB pathway have been shown to promote migration, invasion, and adverse phenotypes in bladder cancer models. These links offer a plausible interface between the neuronal/synaptic enrichment and aggressive tumor behavior, while not constituting evidence of histologic neuroendocrine differentiation in our cohort [19,20].

These transcriptomic features partially overlap with programs observed in the consensus neuroendocrine-like (NE-like) class; nonetheless, our data do not constitute evidence of histologic neuroendocrine differentiation, and subtype summaries are treated as exploratory. Clinically, these observations motivate targeted follow-up—correlating synaptic-adhesion readouts with perineural invasion annotations where available, and assessing protein-level markers (e.g., L1CAM) in tissue microarrays—to clarify whether synaptic-adhesion signaling marks aggressive phenotypes or therapeutic liabilities in BLCA.

In aggregate, the analyses indicate over-representation of Pb-responsive biology within BLCA transcriptomes—most prominently MAPK and cell-cycle programs—and clinically relevant associations between a Pb-response composite signal/AQP12B and overall survival under multivariable adjustment. Important uncertainties remain: the datasets lack individual-level Pb exposure measurements; orthogonal verification at the protein and phospho-signaling level (e.g., ERK1/2, COX-2) is limited; and subtype overlays are exploratory by design. Priority next steps include (i) evaluation in exposure-annotated BLCA cohorts using blood/urine Pb biomarkers, (ii) tissue-level validation with immunohistochemistry or phospho-proteomics on microarrays, and (iii) prospective or nested case-control designs to test causal pathways and therapeutic tractability.

At the level of developmental and stress-inflammation signaling, Notch shows context-dependent roles in cancer and recurrent dysregulation in bladder biology, with documented crosstalk to NF-κB and other stress pathways that may modulate tumor behavior [59]. In this context, recent reviews emphasize that Notch–NF-κB interplay yields context-dependent outcomes in cancer and can intersect with immune regulation and proliferation within the tumor microenvironment [60]. Our pathway summaries—including neuronal/synaptic adhesion and transmission programs—are consistent with contemporary Reactome pathway organization and with modern reappraisals of BLCA signaling dependencies (https://reactome.org/content/detail/R-HSA-5673001?utm_source/, accessed on 14 September 2025).

Finally, occupational cohort data indicate that long-term exposure to straight metalworking fluids is associated with increased bladder cancer risk, underscoring that environmental and workplace Pb exposures remain clinically relevant in modern populations. MWFs are complex mixtures; isolating lead-specific effects is challenging [61]. A population-based study from Poland also reported significantly elevated lead levels in bladder cancer patients, reinforcing the global nature of this risk factor [41]. Methodologically, two aspects warrant emphasis: CTD is a mature, the literature-curated resource that normalizes chemical–gene–disease relationships under controlled vocabularies and continues to expand curation breadth and tooling; meanwhile, UCSC Xena provides public, harmonized TCGA matrices and a curated survival resource that facilitates transparent, reproducible clinico-genomic analyses [14]. See also a peer-reviewed description of Xena’s architecture and data model [39].

Within this framework, our analyses indicate that BLCA tumors are non-randomly enriched for genes previously reported to respond to lead exposure, and that an aggregated lead-response score captures prognostic signal beyond single-gene effects across stage- and sex-defined strata—albeit with modest effect sizes and imprecision in smaller subgroups—when evaluated on harmonized TCGA/Xena data. Causal attribution cannot be made from cross-sectional tumor transcriptomes alone, but the convergence of toxicological, epigenetic, and oncogenic-pathway evidence supports the biological plausibility that lead-responsive programs intersect bladder cancer biology and motivates external validation and mechanistic dissection in controlled exposure models and longitudinal cohorts [49]. Moreover, the prognostic role of *COX-2* in BLCA is heterogeneous across studies: some cohorts report associations with poorer survival, whereas others do not support *COX-2* as an independent marker [62].

One of the key strengths of this study is the integration of high-quality, public genomic data from TCGA and curated toxicogenomic resources like the CTD. This approach merges large-scale transcriptomic data with environmental exposure information, offering insights into the potential links between lead exposure and BLCA biology. By leveraging these publicly available resources, we ensure reproducibility and transparency while contributing to the growing body of research focused on the environmental drivers of cancer.

Additionally, the use of harmonized datasets (TCGA and Xena) allows for the incorporation of diverse patient demographics, ensuring that the findings remain relevant across different subgroups. This broad representation enhances the generalizability of the study’s conclusions. Furthermore, the application of an aggregated lead-response score provides a more comprehensive view of the cumulative effects of lead exposure, moving beyond individual gene analysis, which can be subject to noise or outliers. The study’s methodological framework, which combines epigenetic analysis with genomic data, offers a novel perspective on how toxicant-induced transcriptional shifts may persist and interact with cancer pathways. This integrative approach holds significant promise for identifying new biomarkers for early cancer detection and could pave the way for personalized prevention strategies in populations with environmental exposures.

However, there are several limitations to the current study. The use of cross-sectional data from TCGA limits our ability to draw causal conclusions about the relationship between lead exposure and bladder cancer. Longitudinal studies and controlled exposure models are necessary to better understand the temporal dynamics and long-term effects of lead exposure on bladder cancer pathogenesis. Additionally, although our findings indicate a strong association between lead-responsive genes and bladder cancer, the imprecision observed in smaller subgroups suggests that there must be larger, independent validation cohorts. Furthermore, while we focused on genetic and transcriptomic data, integrating other levels of biological information, such as proteomic and metabolomic data, could provide a more comprehensive understanding of the molecular mechanisms underlying lead-induced carcinogenesis in bladder cancer.

Future studies should aim to validate our findings in prospective cohorts and explore the functional roles of lead-responsive genes in bladder cancer progression. These studies could use more advanced in vitro and in vivo models to assess the direct effects of lead exposure on cell signaling, DNAm, and other epigenetic modifications. Additionally, the potential for lead exposure to interact with other environmental factors, such as smoking or industrial pollutants, should be explored to better understand their combined effects on bladder cancer risk. Another important avenue for future research is the development of lead exposure biomarkers based on DNAm signatures, which could be used for early detection and monitoring of lead-induced carcinogenesis.

This study is an observational secondary analysis of TCGA-BLCA, and does not establish causality. Patient-level lead exposure was not measured; toxicogenomic proxies may not capture exposure timing, dose, or chemical speciation. Available clinical covariates (age, sex and pathological stage) do not include key risk factors such as smoking, treatment, or occupational history, so residual confounding is possible. Event counts were limited for some endpoints (e.g., DFI, DSS), yielding wider CIs and reduced power relative to OS. Database and ontology features (e.g., aggregation within CTD and pathway redundancy) can introduce annotation bias despite HGNC mapping, term-size filtering, and within-collection FDR control. KM plots used median dichotomization for visualization, whereas inference relied on continuous predictors in Cox models. Complete-case analysis avoids imputation, but may introduce selection if missingness is informative. The TCGA case mix (predominantly MIBC) limits generalizability and emphasizes that there must be external validation.

Lastly, therapeutic interventions targeting lead-responsive signaling pathways, such as MAPK or COX-2, could be investigated in preclinical models of bladder cancer to determine if they hold promise for improving patient outcomes. These studies would not only help us better understand the carcinogenic effects of lead, but could also pave the way for novel therapeutic strategies in the treatment of bladder cancer. Consensus molecular labels were developed for MIBC and should be interpreted cautiously in exploratory overlays; accordingly, subtype-stratified outputs are presented descriptively and interpreted with caution to avoid over-interpretation [16]. Finally, while these analyses directly interrogate enrichment and clinical association, they do not establish causal mediation by Pb exposure; dedicated exposure-linked cohorts and experimental validation are required to close this gap.

## 5. Conclusions

Lead-annotated genes curated in CTD are non-randomly over-represented among transcriptional alterations in TCGA BLCA, with pathway enrichments converging on synaptic/adhesion modules, MAPK-centered signaling, and cell-cycle programs. *AQP12B* surfaced as a candidate prognostic marker, although external validation is lacking, and a composite lead-response score showed directionally favorable—although adjusted non-significant—associations with OS and consistent tendencies across subgroups. The aggregate evidence supports biological plausibility for an intersection between lead-responsive pathways and urothelial tumor biology, but falls short of causal inference in humans. Prior epidemiologic studies suggest increased overall and cancer mortality with lead exposure, while bladder-specific risks remain uncertain; embedding quantitative exposure measures and external replication into future designs will be critical. Mechanistic experiments in urothelial systems and class-aware clinical analyses should test whether lead-responsive pathways affect disease progression or therapeutic response.

## Figures and Tables

**Table 1:** Baseline demographics and clinical characteristics of the TCGA-BLCA analytic tumor cohort (N = 412). Values are n (%) for categorical variables and median (IQR) for continuous variables. For survival endpoints, N/events and median follow-up (months) [IQR] are reported for OS (primary) and PFI/DFI/DSS (secondary).

Characteristic	Value
N (tumor cohort)	412
Age, years (median [IQR])	69 [60–76]
Sex—Male	304 (73.8%)
Sex—Female	108 (26.2%)
Pathological stage—I	2 (0.5%)
Pathological stage—II	131 (31.8%)
Pathological stage—III	141 (34.2%)
Pathological stage—IV	136 (33.0%)
Pathological stage—Missing	2 (0.5%)
OS (N/events; median months [IQR])	411/180; 17.6 [10.8–31.6]
PFI (N/events; median months [IQR])	412/177; 14.1 [7.1–26.2]
DFI (N/events; median months [IQR])	189/32; 19.0 [12.2–41.7]
DSS (N/events; median months [IQR])	411/123; 17.6 [10.8–31.6]

BLCA, TCGA bladder urothelial carcinoma; OS, overall survival; PFI, progression-free interval; DFI, disease-free interval; DSS, disease-specific survival; IQR, interquartile range.

**Table 2:** Top 10 most significant DEGs in TCGA BLCA (tumor vs. normal), ranked by FDR. Positive log_2_FC indicates higher expression in tumor; negative values indicate higher expression in normal tissue. Columns: Gene, log_2_FC, *p*-value, FDR.

Gene	log_2_FC	*p*-Value	FDR
*CST4*	1.676252	2.45 × 10^−42^	4.19 × 10^−38^
*PAEP*	1.252275	5.14 × 10^−35^	4.4 × 10^−31^
*SPDYC*	1.244989	8.1 × 10^−33^	4.62 × 10^−29^
*CST2*	1.568127	1.03 × 10^−31^	4.42 × 10^−28^
*AQP12B*	1.16774	6.07 × 10^−30^	2.08 × 10^−26^
*FXYD1*	−1.18892	3.29 × 10^−24^	9.38 × 10^−21^
*TNNI3*	1.497404	2.64 × 10^−23^	6.47 × 10^−20^
*ADH1B*	−1.27773	9.12 × 10^−23^	1.95 × 10^−19^
*C16orf89*	−1.41286	1.88 × 10^−22^	3.57 × 10^−19^
*IL1F7*	0.962661	9.21 × 10^−22^	1.58 × 10^−18^

Note: For the exploratory 10-gene survival screen, both raw and BH-adjusted *p*-values (q-values) across the 10 comparisons are reported; primary interpretation relies on q-values. log_2_FC, log_2_ fold-change; FDR, false discovery rate.

**Table 3:** Log-rank *p*-values for OS across the top 10 DEGs in TCGA BLCA.

Gene	N_high	N_low	Logrank_p_OS	Cox_HR_ per_SD_OS	Cox_95%CI_low	Cox_95%CI_high	Cox_p_OS	PFI_N	PFI_logrank_p	DFI_N	DFI_logrank_p
*AQP12B*	203	203	0.004	0.771	0.644	0.923	0.004	407	0.023	187	0.121
*C16orf89*	203	203	0.015	1.116	0.971	1.282	0.121	407	0.134	187	0.693
*CST4*	203	203	0.134	0.865	0.735	1.019	0.083	407	0.136	187	0.764
*FXYD1*	203	203	0.142	1.126	0.977	1.297	0.099	407	0.354	187	0.806
*SPDYC*	203	203	0.190	1.115	0.979	1.270	0.099	407	0.397	187	0.300
*TNNI3*	203	203	0.190	1.038	0.895	1.203	0.618	407	0.198	187	0.430
*CST2*	203	203	0.286	1.127	0.976	1.301	0.100	407	0.856	187	0.290
*PAEP*	203	203	0.395	1.078	0.932	1.247	0.310	407	0.168	187	0.593
*ADH1B*	203	203	0.451	1.070	0.928	1.235	0.348	407	0.212	187	0.982
*IL1F7*	203	203	0.814	0.925	0.782	1.094	0.364	407	0.835	187	0.566

Note: KM median split is exploratory and shown for visualization; interpretation relies on continuous (per SD) Cox estimates. For OS, PFI, and DFI, reported N values reflect endpoint-specific complete cases after harmonizing curated clinical data with gene-level expression. In KM displays, N corresponds to the total of the two median-split strata among cases with non-missing endpoint and gene values; due to per-gene missingness after merges, totals may be smaller than the overall analytic cohort.

**Table 4:** Multivariable Cox models (age/sex/stage-adjusted) for OS, and where available PFI, DFI, and DSS in TCGA BLCA.

Gene	Endpoint	N	HR_gene_perSD	CI_low	CI_high	p_Wald
*CST4*	DFI	186	0.762	0.506	1.147	0.193
*AQP12B*	DFI	186	0.750	0.486	1.157	0.194
*IL1F7*	DFI	186	0.804	0.530	1.217	0.303
*FXYD1*	DFI	186	0.844	0.581	1.225	0.374
*C16orf89*	DFI	186	0.851	0.576	1.258	0.421
*TNNI3*	DFI	186	0.877	0.596	1.290	0.506
*PAEP*	DFI	186	1.079	0.763	1.525	0.665
*CST2*	DFI	186	0.936	0.637	1.373	0.735
*SPDYC*	DFI	186	1.050	0.750	1.469	0.775
*ADH1B*	DFI	186	0.999	0.692	1.443	0.998
*AQP12B*	DSS	391	0.710	0.560	0.899	0.004
*CST4*	DSS	391	0.799	0.645	0.990	0.040
*IL1F7*	DSS	391	0.855	0.684	1.070	0.172
*PAEP*	DSS	391	1.082	0.906	1.292	0.380
*SPDYC*	DSS	391	1.071	0.908	1.263	0.411
*ADH1B*	DSS	391	0.961	0.804	1.148	0.662
*TNNI3*	DSS	391	0.965	0.806	1.154	0.697
*C16orf89*	DSS	391	1.028	0.862	1.225	0.756
*FXYD1*	DSS	391	0.981	0.818	1.176	0.836
*CST2*	DSS	391	0.981	0.817	1.177	0.839
*AQP12B*	OS	404	0.761	0.633	0.915	0.003
*CST4*	OS	404	0.841	0.708	1.000	0.050
*IL1F7*	OS	404	0.874	0.731	1.044	0.139
*SPDYC*	OS	404	1.089	0.952	1.245	0.211
*C16orf89*	OS	404	1.082	0.939	1.247	0.273
*PAEP*	OS	404	1.075	0.926	1.248	0.336
*CST2*	OS	404	1.023	0.879	1.190	0.765
*FXYD1*	OS	404	1.020	0.879	1.184	0.790
*TNNI3*	OS	404	0.980	0.846	1.136	0.795
*ADH1B*	OS	404	1.001	0.865	1.159	0.983
*CST4*	PFI	405	0.809	0.682	0.960	0.015
*PAEP*	PFI	405	1.140	0.988	1.316	0.070
*AQP12B*	PFI	405	0.853	0.716	1.017	0.077
*IL1F7*	PFI	405	0.870	0.726	1.042	0.130
*CST2*	PFI	405	0.945	0.810	1.104	0.481
*FXYD1*	PFI	405	0.947	0.816	1.101	0.484
*C16orf89*	PFI	405	0.954	0.821	1.109	0.546
*TNNI3*	PFI	405	1.030	0.888	1.195	0.690
*SPDYC*	PFI	405	1.028	0.890	1.187	0.700
*ADH1B*	PFI	405	0.992	0.855	1.150	0.917

Columns: Gene; Endpoint; N; HR per 1 SD; 95% CI; Wald p. HR, hazard ratio; CI, confidence interval; OS, overall survival; PFI, progression-free interval; DFI, disease-free interval; DSS, disease-specific survival.

**Table 5:** Representative subset of the intersection between BLCA DEGs (FDR < 0.05) and lead-associated genes from CTD. The table reports the top 15 upregulated and top 15 downregulated genes prioritized by FDR (ties broken by |log_2_FC|). Columns: Gene, Direction (Up/Down), log_2_FC, FDR. The complete intersection is reported in Supplementary Table S5.

Gene	Direction	log_2_FC	FDR
*SNORA72*	Up	0.459	3.39 × 10^−20^
*TNNI3*	Up	2.45	4.52 × 10^−20^
*SERPINA10*	Up	0.45	4.41 × 10^−17^
*SMPD4*	Up	0.671	8.35 × 10^−16^
*AHSG*	Up	0.823	2.31 × 10^−15^
*ORM2*	Up	0.658	8.56 × 10^−15^
*KNG1*	Up	0.59	3.32 × 10^−13^
*ILF3*	Up	0.579	5.54 × 10^−11^
*XPO5*	Up	0.764	3.05 × 10^−10^
*FOXK2*	Up	0.575	3.15 × 10^−10^
*APCS*	Up	0.383	8.81 × 10^−10^
*GC*	Up	0.756	9.97 × 10^−10^
*ALB*	Up	1.576	1.02 × 10^−09^
*CCT6A*	Up	0.537	7.59 × 10^−09^
*ACP1*	Up	0.65	9.97 × 10^−09^
*FXYD1*	Down	−5.271	6.75 × 10^−21^
*ADH1B*	Down	−7.567	1.34 × 10^−19^
*SOX10*	Down	−4.782	1.87 × 10^−18^
*CADM3*	Down	−5.809	6.06 × 10^−18^
*BCL2*	Down	−2.033	4.44 × 10^−16^
*SYBU*	Down	−2.374	8.61 × 10^−15^
*F10*	Down	−4.712	9.37 × 10^−15^
*PHYHIP*	Down	−3.931	1.09 × 10^−13^
*RCBTB2*	Down	−1.306	1.96 × 10^−13^
*NRXN1*	Down	−5.583	3.50 × 10^−13^
*GFRA1*	Down	−4.808	9.58 × 10^−13^
*PTH1R*	Down	−3.298	9.58 × 10^−13^
*SORCS1*	Down	−5.391	1.53 × 10^−12^
*RASGEF1C*	Down	−2.593	1.72 × 10^−12^
*MATN2*	Down	−3.231	1.98 × 10^−12^

**Table 6:** Top enriched pathways for the Lead–DEG overlap in TCGA BLCA (GO BP and Reactome, top 20 each). Columns: collection, term (lowercase), k/n (GeneRatio), K (term size), p (hypergeometric), FDR (BH).

Collection	Term	k/n	K	*p*	FDR
GO BP	Gobp regulation of trans synaptic signaling	0.0627	483	6.17 × 10^−20^	2.95 × 10^−16^
GO BP	Gobp cognition	0.0420	308	7.36 × 10^−15^	2.95 × 10^−16^
GO BP	Gobp positive regulation of synaptic transmission	0.0282	158	7.62 × 10^−15^	2.95 × 10^−16^
GO BP	Gobp regulation of nervous system development	0.0514	424	8.75 × 10^−15^	2.95 × 10^−16^
GO BP	Gobp axon development	0.0571	499	1.19 × 10^−14^	2.95 × 10^−16^
GO BP	Gobp regulation of synaptic plasticity	0.0320	204	4.03 × 10^−14^	2.95 × 10^−16^
GO BP	Gobp chromosome organization	0.0527	475	7.28 × 10^−13^	2.95 × 10^−16^
GO BP	Gobp positive regulation of nervous system development	0.0357	264	1.06 × 10^−12^	2.95 × 10^−16^
GO BP	Gobp developmental maturation	0.0408	332	3.06 × 10^−12^	2.95 × 10^−16^
GO BP	Gobp neurotransmitter transport	0.0301	208	5.27 × 10^−12^	2.95 × 10^−16^
GO BP	Gobp gliogenesis	0.0395	327	1.37 × 10^−11^	2.95 × 10^−16^
GO BP	Gobp regulation of membrane potential	0.0483	444	1.80 × 10^−11^	2.95 × 10^−16^
GO BP	Gobp regulation of monoatomic ion transport	0.0451	403	1.99 × 10^−11^	2.95 × 10^−16^
GO BP	Gobp regulation of transmembrane transport	0.0433	388	6.49 × 10^−11^	2.95 × 10^−16^
GO BP	Gobp neuron apoptotic process	0.0357	297	1.50 × 10^−10^	2.95 × 10^−16^
GO BP	Gobp cell junction assembly	0.0502	492	1.92 × 10^−10^	2.95 × 10^−16^
GO BP	Gobp cell growth	0.0470	453	3.07 × 10^−10^	2.95 × 10^−16^
GO BP	Gobp regulation of neurogenesis	0.0389	349	6.31 × 10^−10^	2.95 × 10^−16^
GO BP	Gobp long term synaptic potentiation	0.0176	97	6.43 × 10^−10^	2.95 × 10^−16^
GO BP	Gobp regulation of DNA metabolic process	0.0445	427	7.56 × 10^−10^	2.95 × 10^−16^
Reactome	Neuronal system	0.0520	407	2.44 × 10^−16^	3.77 × 10^−13^
Reactome	Cell cycle, mitotic	0.0470	452	2.77 × 10^−10^	3.77 × 10^−13^
Reactome	MAPK1/MAPK3 signaling	0.0307	264	9.07 × 10^−09^	3.77 × 10^−13^
Reactome	Mapk family signaling cascades	0.0332	299	1.18 × 10^−08^	3.77 × 10^−13^
Reactome	Diseases of signal transduction by growth factor receptors and second messengers	0.0433	438	1.29 × 10^−08^	3.77 × 10^−13^
Reactome	Intracellular signaling by second messengers	0.0326	294	1.75 × 10^−08^	3.77 × 10^−13^
Reactome	Neurexins and neuroligins	0.0119	57	2.70 × 10^−08^	3.77 × 10^−13^
Reactome	G1/s transition	0.0176	114	3.29 × 10^−08^	3.77 × 10^−13^
Reactome	Mitotic g1 phase and g1/s transition	0.0188	132	7.12 × 10^−08^	3.77 × 10^−13^
Reactome	L1cam interactions	0.0169	116	1.89 × 10^−07^	3.77 × 10^−13^
Reactome	Cell cycle checkpoints	0.0257	231	5.02 × 10^−07^	3.77 × 10^−13^
Reactome	DNA repair	0.0282	267	6.47 × 10^−07^	3.77 × 10^−13^
Reactome	M phase	0.0313	315	1.01 × 10^−06^	3.77 × 10^−13^
Reactome	Dopamine neurotransmitter release cycle	0.0063	22	2.11 × 10^−06^	3.77 × 10^−13^
Reactome	Base-excision repair, ap site formation	0.0056	18	2.57 × 10^−06^	3.77 × 10^−13^
Reactome	Glutamate neurotransmitter release cycle	0.0063	23	3.48 × 10^−06^	3.77 × 10^−13^
Reactome	Base excision repair	0.0088	45	4.62 × 10^−06^	3.77 × 10^−13^
Reactome	Adherens junctions interactions	0.0194	168	5.24 × 10^−06^	3.77 × 10^−13^
Reactome	G-protein mediated events	0.0094	53	7.86 × 10^−06^	3.77 × 10^−13^
Reactome	Acetylcholine neurotransmitter release cycle	0.0050	16	9.53 × 10^−06^	3.77 × 10^−13^

**Table 7:** Lead-response score: Cox survival models.

Endpoint	Model	HR_per_SD	CI_low	CI_high	*p*	N
OS	Univariable	0.902	0.788	1.03	0.138	406
OS	Multivariable (age, sex, stage-ordinal)	0.925	0.812	1.05	0.244	404
PFI	Univariable	0.956	0.832	1.1	0.533	407
PFI	Multivariable (age, sex, stage-ordinal)	0.99	0.865	1.13	0.888	405
DFI	Univariable	1.02	0.707	1.48	0.907	187
DFI	Multivariable (age, sex, stage-ordinal)	1.1	0.747	1.63	0.625	186
DSS	Univariable	0.901	0.765	1.06	0.214	393
DSS	Multivariable (age, sex, stage-ordinal)	0.941	0.803	1.1	0.452	391

HR, hazard ratio; CI, confidence interval; OS, overall survival; PFI, progression-free interval; DFI, disease-free interval; DSS, disease-specific survival.

## Data Availability

Data are contained within the article.

## Supplementary Materials

The following supporting information can be downloaded at: https://www.mdpi.com/article/10.3390/cancers17203291/s1, Table S1: Sensitivity analysis of AQP12B for OS under alternative parameterizations of pathological stage. The primary model treats stage as an ordinal numeric covariate (I–IV → 1–4), whereas the sensitivity model uses categorical indicators (Stage I as reference). Hazard ratios (HRs) are reported per one standard deviation (1-SD) increase in expression and are adjusted for age (per 10 years), sex, and stage. Inference is based on Wald statistics with two-sided α = 0.05; missingness handled via complete-case analysis. Columns: Model; Gene; Endpoint; N; HR per 1-SD; 95% CI; Wald p. Table S2: Stage-indicator coefficients from the categorical-stage Cox model for OS with Stage I as the reference category. Estimates correspond to the incremental effect of Stage II, III, and IV relative to Stage I, in a model additionally adjusted for age (per 10 years), sex, and AQP12B expression (per 1-SD). Wald 95% confidence intervals and two-sided *p*-values are reported; complete-case analysis was used. Columns: Term (Stage II/III/IV vs I); HR; 95% CI (low–high); Wald p; N. Table S3: Multivariable Cox estimates for AQP12B across endpoints in TCGA BLCA. Endpoints include OS, progression-free interval (PFI), disease-free interval (DFI), and disease-specific survival (DSS). Hazard ratios are reported per 1-SD increase in expression, adjusted for age (per 10 years), sex, and pathological stage (I–IV, ordinal). Wald 95% confidence intervals and two-sided *p*-values are provided; complete-case analysis per endpoint. Columns: Endpoint; N; HR per 1-SD; 95% CI; Wald p. Table S4: Full intersection between BLCA DEGs defined by the stricter threshold (FDR < 0.05 and |log_2_FC| ≥ 1) and CTD Lead-associated genes. Counts: DEGs n = 4275; overlap k = 698; right-tailed hypergeometric *p* = 9.86 × 10^−15^. Columns: Gene, Direction (Up/Down), log_2_FC, FDR. Table S5: Full intersection between BLCA DEGs (FDR < 0.05) and CTD Lead-associated genes under the primary threshold. Counts: universe M = 20,530, CTD-Lead K = 2618, DEGs n = 11,436, overlap k = 1595, *p* = 4.21 × 10^−9^ (right-tailed hypergeometric). Columns: Gene, Direction (Up/Down), log_2_FC, FDR. Table S6: Complete over-representation results for the Lead–DEG overlap using GO Biological Process (MSigDB C5, symbols.gmt) and Reactome (symbols.gmt) gene-set collections. Background universe: genes quantified in the TCGA BLCA RNA-seq matrix (UCSC Xena). Columns: term, k (overlap size), n (query size), K (term size), M (universe size), GeneRatio (=k/n), p (right-tailed hypergeometric), FDR (Benjamini–Hochberg), and LeadingGenes (overlapping genes; truncated for display). Separate worksheets summarize GO BP and Reactome. For context, neuronal/synaptic terms (neuronal system, neurexins/neuroligins, L1CAM) are interpreted as synaptic-adhesion/communication programs that may relate to nerve–tumor crosstalk and, at a transcriptomic level, to features seen in the NE-like class; these exploratory links are not claims of histologic neuroendocrine differentiation. Table S7: Stage coding sensitivity (OS). Table S8: Multivariable HRs by endpoint (OS, PFI, DFI, DSS). Table S9: Subgroup Cox models for OS using the lead-response score. Summary of Cox proportional-hazards models reporting the association between the z-standardized lead-response score and OS within pre-specified strata. Columns give Stratum, Model adjustment (Stage I–II and Stage III–IV: age per 10 years; Male and Female: age per 10 years plus pathological stage encoded ordinally I–IV), N (complete-case count), HR per 1-SD, 95% CI (low–high), and two-sided *p*-value. Ties were handled via the Breslow method. Figure S1. Adjusted hazard ratio curve from a Cox model with restricted cubic splines (3 knots) for standardized AQP12B expression in TCGA-BLCA. The solid line denotes the HR relative to z = 0; the shaded band shows the 95% CI. Vertical tick marks indicate the empirical density (rug). The horizontal dashed line marks HR = 1. A likelihood-ratio test for non-linearity is reported on the panel. This display is supportive; primary inference relies on the continuous (per-SD) Cox specification used in the main text. Figure S2: (A): Kaplan–Meier overall survival curves by tertiles of AQP12B expression (Low/Mid/High) in TCGA-BLCA. Group sizes are shown in the legend; the x-axis shows months from diagnosis and the y-axis overall-survival probability. The log-rank *p*-value is reported on the panel. This tertile split is exploratory and intended for visualization only; interpretation is based on continuous Cox estimates. (B): Kaplan–Meier overall survival curves using a cross-validated maximally separated log-rank cut (maxstat) for AQP12B expression. The cutpoint was selected by internal 10-fold cross-validation. Group sizes are shown in the legend; axes as in S2A; the log-rank *p*-value is reported. As an exploratory display, this figure complements the primary continuous Cox modeling. Figure S3: Forest plot summarizing multivariable Cox models for progression-free interval (PFI) in TCGA BLCA. Points denote hazard ratios per 1-SD increase in gene expression; horizontal bars indicate Wald 95% confidence intervals. All models are adjusted for age (per 10 years), sex, and pathological stage (I–IV, ordinal). The vertical dashed line marks HR = 1; lower HR indicates association with longer PFI. Figure S4: Subgroup forest plot for overall survival (OS) by the lead-response score in TCGA BLCA. Forest plot showing hazard ratios (HRs) per one standard deviation (SD) increase in the lead-response gene-set score across pre-specified strata: Stage I–II (age-adjusted), Stage III–IV (age-adjusted), male (adjusted for age and pathological stage), and female (adjusted for age and pathological stage). Points denote HR estimates and horizontal bars indicate Wald 95% confidence intervals (CIs); the vertical dashed line marks HR  =  1. Models are Cox proportional hazards with Breslow handling of ties; the score was z-standardized across tumors and constructed from the overlap between CTD Lead–associated genes and BLCA differentially expressed genes. Figure S5: Pb → Mechanism Map (conceptual schematic summarizing ROS↑, MAPK/ERK and p38 activation, EGFR transactivation, COX-2 induction, and ancillary effects on DNA repair, mitochondria, and epigenetic reprogramming; with subtype context notes for Basal/Squamous and Luminal Papillary).

## Abbreviations

BC, bladder cancer; BLCA, TCGA bladder cancer project; TCGA, The Cancer Genome Atlas; GDC, Genomic Data Commons; UCSC Xena, University of California, Santa Cruz Xena platform; IARC, International Agency for Research on Cancer; CTD, Comparative Toxicogenomics Database; EWAS, epigenome-wide association study; DNAm, DNA methylation; MIBC, muscle-invasive bladder cancer; NMIBC, non–muscle-invasive bladder cancer; RTK, receptor tyrosine kinase; RAS, rat sarcoma family; MAPK, mitogen-activated protein kinase; PI3K, phosphatidylinositol 3-kinase; TP53/RB1, tumor suppressors TP53 and RB1; FGFR, fibroblast growth factor receptor; EGFR, epidermal growth factor receptor; COX-2, cyclooxygenase-2; PAH(s), polycyclic aromatic hydrocarbon(s); Pb, lead; DE, differential expression; DEG(s), differentially expressed gene(s); ORA, over-representation analysis; GO BP, Gene Ontology—Biological Process; FDR, false discovery rate; BH, Benjamini–Hochberg; HR, hazard ratio; CI, confidence interval; SD, standard deviation; KM, Kaplan–Meier; OS, overall survival; PFI, progression-free interval; DFI, disease-free interval; DSS, disease-specific survival; LOO, leave-one-out;
